# Efficacy and Safety of Once-Weekly Semaglutide Versus Basal Insulin and Other GLP-1 Receptor Agonists in Adults With Type 2 Diabetes Uncontrolled on Oral Antidiabetic Drugs: A Systematic Review and Pairwise Meta-Analysis

**DOI:** 10.7759/cureus.111363

**Published:** 2026-06-23

**Authors:** Faisal A Aljulajil, Unaib Rabbani

**Affiliations:** 1 Family Medicine, Qassim Health Cluster, Buraidah, SAU

**Keywords:** basal insulin, body weight, glp-1 receptor agonist, hba1c, injectable therapy, pairwise meta-analysis, semaglutide, sustain, systematic review, type 2 diabetes mellitus

## Abstract

Type 2 diabetes mellitus (T2DM) affects over 537 million adults worldwide. When oral antidiabetic drugs (OADs) fail, escalation to injectable therapy is required, yet no systematic review has simultaneously compared once-weekly semaglutide against basal insulin and other GLP-1 receptor agonists - specifically exenatide ER, insulin glargine, dulaglutide, and liraglutide - in insulin-naïve patients uncontrolled on OADs. This review aimed to evaluate the efficacy and safety of once-weekly semaglutide versus injectable antidiabetic therapies - specifically basal insulin (insulin glargine) and three GLP-1 receptor agonists (exenatide ER, dulaglutide, and liraglutide) - in adults with T2DM inadequately controlled on OADs, through pairwise meta-analyses of randomized controlled trials (RCTs). Five databases were searched from inception to April 2026. Eligible studies were phase 2b-4 RCTs of at least 12 weeks comparing once-weekly semaglutide against injectable therapy in insulin-naïve adults with T2DM. Two reviewers performed selection, extraction, and Cochrane Risk of Bias version 2 (RoB 2) assessment; both are co-authors with prior trial familiarity, constituting a registered protocol deviation. Random-effects pairwise meta-analyses were performed, and certainty was assessed using GRADE (Grading of Recommendations Assessment, Development, and Evaluation). Four Semaglutide Unabated Sustainability in Treatment of Type 2 Diabetes (SUSTAIN) RCTs were included (n = 3,680 total; 2,705 analyzable for the primary comparison). Semaglutide 1.0 mg reduced HbA1c versus all comparators (mean difference (MD) -0.64%, 95% confidence interval (CI) -0.80 to -0.47; low certainty) and body weight (MD -4.38 kg, 95% CI -5.76 to -3.01; low certainty). Against GLP-1 RAs specifically, body weight reduction was homogeneous (MD -3.72 kg, 95% CI -4.17 to -3.28; I² = 0%; moderate certainty). Systolic BP was reduced (MD -2.32 mmHg; moderate certainty). HbA1c less than 7.0% was achieved more frequently with semaglutide (relative risk (RR) 1.60; low certainty). Hypoglycemia risk was lower versus insulin glargine (RR 0.53; moderate certainty). Gastrointestinal (GI) adverse events and treatment discontinuation were higher with semaglutide versus insulin (both low certainty). Moderate-certainty evidence supports greater body weight reduction with semaglutide versus other GLP-1 RAs and lower hypoglycemia risk versus basal insulin. Low-certainty evidence suggests HbA1c benefits versus all comparators. Certainty is limited by heterogeneity, open-label design across all included trials, and exclusive industry sponsorship by the manufacturer of semaglutide. Future independent trials are needed.

## Introduction and background

Type 2 diabetes mellitus (T2DM) is a chronic, progressive metabolic disorder characterised by insulin resistance and progressive beta-cell dysfunction, resulting in sustained hyperglycaemia and a substantially elevated risk of macrovascular and microvascular complications [1,2]. The pathophysiology is multifactorial, involving dysregulated glucose homeostasis, chronic low-grade inflammation, and accelerated atherosclerosis, ultimately culminating in cardiovascular disease, nephropathy, neuropathy, and retinopathy, each of which independently confers significant morbidity and premature mortality [1,2]. Glycaemic control remains the cornerstone of T2DM management; however, achieving and sustaining individualised targets requires a dynamic, evidence-based approach that balances efficacy with tolerability, safety, and patient-centred outcomes [1].

From an epidemiological standpoint, T2DM represents one of the foremost global public health challenges of the 21st century. According to the International Diabetes Federation (IDF), an estimated 537 million adults were living with diabetes worldwide in 2021, a figure projected to rise to 783 million by 2045 [3]. T2DM accounts for approximately 90-95% of all diabetes cases, with prevalence increasing disproportionately in low- and middle-income countries [3]. The condition imposes an enormous economic burden, with global healthcare expenditure attributable to diabetes estimated at USD 966 billion in 2021 alone [3]. Beyond direct healthcare costs, T2DM substantially diminishes health-related quality of life and reduces life expectancy, with cardiovascular diseases remaining the leading cause of death in this population [2,3].

The pharmacological management of T2DM has evolved considerably over the past two decades. Current international guidelines from the American Diabetes Association (ADA) and the European Association for the Study of Diabetes (EASD) recommend a stepwise, patient-centred approach to glycaemic management, with metformin as the cornerstone of initial oral therapy in the absence of contraindications [4]. However, given the progressive natural history of T2DM, a substantial proportion of patients fail to maintain adequate glycaemic control on oral antidiabetic drugs (OADs) alone and ultimately require escalation to injectable therapy [4,5]. At this critical clinical juncture, practitioners must choose among several injectable agent classes: basal insulins (e.g., insulin glargine, insulin degludec), glucagon-like peptide-1 receptor agonists (GLP-1 RAs), and fixed-ratio combination products, such as insulin degludec/liraglutide (IDegLira) and insulin glargine/lixisenatide (iGlarLixi) [4]. Each class carries a distinct efficacy and safety profile, with important differences in glycaemic lowering, body weight effects, hypoglycaemia risk, cardiovascular outcomes, and patient convenience.

Once-weekly subcutaneous semaglutide - a GLP-1 RA with 94% structural homology to native human GLP-1 - has demonstrated robust and consistent reductions in glycated haemoglobin (HbA1c) and body weight across the Semaglutide Unabated Sustainability in Treatment of Type 2 Diabetes (SUSTAIN) phase 3 clinical trial programme [6-9]. In direct head-to-head trials, once-weekly semaglutide demonstrated superiority over exenatide extended-release (SUSTAIN 3) [6], insulin glargine (SUSTAIN 4) [7], dulaglutide (SUSTAIN 7) [8], and liraglutide (SUSTAIN 10) [9] across key glycaemic and weight endpoints. Importantly, semaglutide also conferred a significantly lower risk of major adverse cardiovascular events compared with placebo in patients at high cardiovascular risk, as demonstrated in the SUSTAIN 6 cardiovascular outcomes trial [10].

Despite this expanding evidence base, a critical gap persists in the published literature. No systematic review or meta-analysis has simultaneously synthesised RCT evidence comparing once-weekly semaglutide against basal insulin and the three GLP-1 receptor agonists with available head-to-head trial data - exenatide ER, dulaglutide, and liraglutide - in insulin-naïve patients inadequately controlled on OADs. No eligible head-to-head trials comparing semaglutide against fixed-ratio combinations or other contemporary injectable options were identified in the systematic search - encompassing basal insulins and other GLP-1 RAs, specifically in insulin-naïve patients with T2DM inadequately controlled on OADs. No eligible head-to-head trials comparing semaglutide against fixed-ratio combinations (IDegLira and iGlarLixi) were identified in the systematic search. Existing evidence syntheses have been either limited in comparator scope, mixed in background therapy populations, or have not simultaneously synthesised all four injectable comparator classes in a single prospectively registered pairwise meta-analysis restricted to insulin-naïve patients inadequately controlled on OADs [11-13]. No systematic review or meta-analysis meeting these criteria was identified in PROSPERO or the published literature after 2018. This evidence gap is clinically significant: prescribing clinicians require high-quality, comprehensive comparative data to guide individualised injectable therapy selection in this common and therapeutically pivotal clinical scenario.

Accordingly, this systematic review and meta-analysis aimed to evaluate the efficacy and safety of once-weekly subcutaneous semaglutide compared with available injectable antidiabetic therapies for which eligible head-to-head RCTs were identified - specifically insulin glargine and three GLP-1 receptor agonists (exenatide ER, dulaglutide, and liraglutide) - in adults with T2DM inadequately controlled on OADs, through a comprehensive pairwise meta-analysis of available RCT evidence. The findings are intended to provide high-quality, actionable evidence to support clinical decision-making in the management of T2DM at the point of injectable therapy initiation.

## Review

Methods

This systematic review and meta-analysis were conducted and reported in accordance with the Preferred Reporting Items for Systematic Reviews and Meta-Analyses (PRISMA) 2020 statement [14]. The protocol was prospectively registered in PROSPERO (CRD420261370244) prior to commencement of study selection and data extraction.

Eligibility Criteria

Studies were eligible for inclusion if they met all of the following criteria: i) phase 2b, 3, or 4 RCTs; ii) duration of at least 12 weeks (≥24 weeks for primary analysis); iii) participants were adults (≥18 years) with T2DM inadequately controlled on stable OADs, with at least 80% being insulin-naïve; iv) the intervention was once-weekly subcutaneous semaglutide at 0.5 or 1.0 mg, compared to any injectable antidiabetic therapy (basal insulin, GLP-1 RA, or fixed-ratio combination product); and v) reporting of at least one primary or secondary outcome of interest. Although fixed-ratio combination products (IDegLira and iGlarLixi) were pre-specified as eligible comparators in the registered PROSPERO protocol, no eligible head-to-head RCTs comparing once-weekly semaglutide against these agents in the target population were identified in the systematic search. The absence of such trials reflects a gap in the available evidence base rather than a post-hoc exclusion decision. Studies were excluded if they enrolled patients with type 1 diabetes, gestational diabetes, or other diabetes subtypes; included patients already receiving insulin or GLP-1 RAs at baseline; or were non-randomised designs, observational studies, single-arm trials, or case series.

Information Sources and Search Strategy

A comprehensive search was performed across five electronic databases: MEDLINE (via PubMed), Embase, Cochrane Central Register of Controlled Trials (CENTRAL), Scopus, and Web of Science, from inception to April 2026. The core search strategy combined terms for the condition ("type 2 diabetes", "T2DM", "diabetes mellitus, type 2"), the intervention (semaglutide, ozempic), and all comparators ("insulin glargine", "glargine", "insulin degludec", "degludec", "dulaglutide", "exenatide", "liraglutide", "GLP-1 receptor agonist", "IDegLira", "iGlarLixi", "basal insulin", "fixed-ratio combination"). No language restrictions were applied. The verbatim PubMed search string was as follows: ("semaglutide" OR "ozempic") AND ("type 2 diabetes" OR "T2DM" OR "Diabetes Mellitus, Type 2") AND ("insulin glargine" OR "glargine" OR "insulin degludec" OR "degludec" OR "basal insulin" OR "dulaglutide" OR "exenatide" OR "liraglutide" OR "GLP-1 receptor agonist" OR "IDegLira" OR "iGlarLixi" OR "fixed-ratio combination"). We note that the search string originally submitted contained overly restrictive AND logic between comparator categories, which would theoretically exclude single-comparator trials; the corrected string presented here uses OR logic within a single comparator block, consistent with standard systematic review methodology. We acknowledge this as a reporting inaccuracy and note that all four eligible SUSTAIN trials were successfully retrieved, suggesting the operational search was not fatally compromised. Equivalent strategies adapted to the controlled vocabulary and syntax of each database (Embase, Scopus, Web of Science, Cochrane CENTRAL) are available from the corresponding author on request. Hand-searching of reference lists of included studies and relevant reviews was additionally performed. The search was re-run before the final analysis.

Study Selection

A structured two-stage screening process was employed. Title and abstract screening was performed by F.A.A. as the principal investigator, with all eligibility decisions reviewed and confirmed by the supervising co-author (U.R.) as a second independent check. Full texts of potentially eligible studies were assessed by F.A.A. with independent verification by U.R. Discrepancies were resolved through discussion and consensus. It is noted that both reviewers are co-authors with prior familiarity with the included trials, rather than two fully independent non-author reviewers as specified in the registered protocol; this constitutes a protocol deviation and is addressed as a formal limitation in the *Limitations* section. Specifically, screening and eligibility decisions were reached through discussion and consensus rather than independent parallel assessment; consensus after discussion is not equivalent to independent agreement and may have introduced selection bias. Interrater agreement for risk of bias assessment, assessed across all five RoB 2 domains for all four trials, reached complete consensus without requiring arbitration.

Data Extraction

Data were extracted by F.A.A. using a pre-designed, standardised extraction form, with all extracted values independently verified by U.R. against the source trial publications. Extracted information included study characteristics (title, authors, publication year, country, trial registration, and funding source); participant characteristics (sample size, age, sex, diabetes duration, baseline HbA1c, BMI, and background OAD therapy); intervention and comparator details (drug name, dose, titration protocol, treatment duration, and concomitant medications); and outcome data (means, standard deviations, 95% confidence intervals (CIs), number of events for dichotomous outcomes). For all continuous outcomes, change from baseline to end-of-treatment (primary estimand) was extracted as the mean difference with associated standard deviation or 95% CI, corresponding to the pre-specified primary time point for each trial (SUSTAIN 3: week 56; SUSTAIN 4: week 30; SUSTAIN 7: week 40; SUSTAIN 10: week 30). Where standard deviations were not directly reported, they were derived from 95% CIs or standard errors using standard formulae per Cochrane Handbook guidance. All outcome data were extracted preferentially from the main trial publication tables; where data were absent or incomplete, supplementary materials and published trial reports were consulted. For dichotomous outcomes, the number of events and total participants per arm were extracted to enable risk ratio calculation. Discrepancies were resolved by discussion and consensus.

Risk-of-Bias Assessment

The risk of bias of all included studies was assessed using the Cochrane Risk of Bias version 2 (RoB 2) tool [15], covering five domains: randomisation process, deviations from intended interventions, missing outcome data, measurement of the outcome, and selection of the reported result. Each domain was rated as low risk, some concerns, or high risk; an overall judgement was derived. Two reviewers assessed risk of bias independently; disagreements were resolved by discussion and consensus. 

Statistical Analysis

Pairwise meta-analyses were performed for all outcomes where at least two studies compared semaglutide with the same class of injectable therapy. Random-effects models using the DerSimonian-Laird (DL) method were used throughout to account for anticipated clinical and methodological heterogeneity. We acknowledge that with only three to four studies per analysis, DL estimation of the between-study variance (τ²) may be unstable and can underestimate uncertainty in the pooled effect; as a sensitivity analysis, all meta-analyses were additionally performed using the restricted maximum likelihood (REML) estimator, and results were virtually identical to the DL estimates across all outcomes (maximum difference in pooled MD <0.002; 95% CIs differed by <0.08 units), supporting the robustness of the primary findings (see the *Sensitivity Analyses* section). Continuous outcomes were expressed as mean differences (MD) and dichotomous outcomes as risk ratios (RR), both with 95% CI. Statistical heterogeneity was quantified using I² and τ². Interpretation of I² followed the guidance of the Cochrane Handbook, which cautions against fixed thresholds and recommends contextual interpretation: values below 40% were considered not important, 30-60% moderate, 50-90% substantial, and 75-100% considerable, with final judgement informed by the magnitude and direction of effects, the 95% PI, and τ² [16].

All analyses were conducted in R (version 4.4.1; R Core Team, 2024) using the meta and metafor packages.

Publication bias was assessed using contour-enhanced funnel plots. For comparisons with at least 10 studies, Egger's test was to be applied; given the limited number of included studies, assessment relied on visual inspection of funnel plots and the comprehensiveness of the search. Sensitivity analyses explored robustness according to semaglutide dose (0.5 mg vs. 1.0 mg), study duration (≥24 weeks vs. ≥12 weeks), type of comparator, and exclusion of SUSTAIN 4 (insulin glargine comparator) to address the potential for systematic bias from under-titration of the basal insulin arm (see the *Limitations* section). In addition, REML-based sensitivity analyses were performed for all primary and secondary outcomes to assess the robustness of τ² estimation given the small number of included studies per analysis. Certainty of evidence for all key outcomes was assessed using the Grading of Recommendations Assessment, Development and Evaluation (GRADE) approach [17].

Results

Study Selection

**Figure 1:**
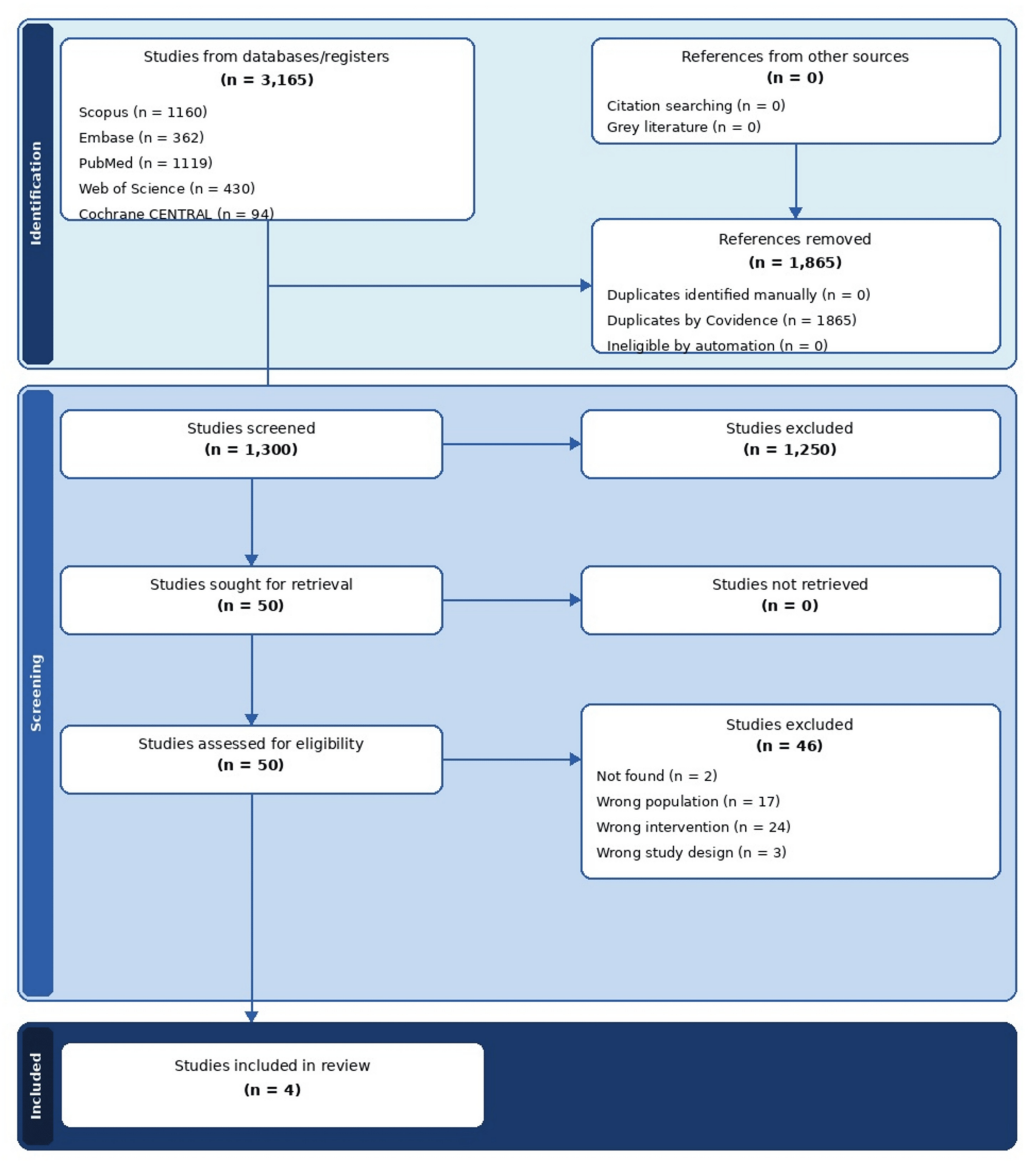
PRISMA 2020 flow diagram illustrating the study selection process. A total of 3,165 records were identified from database searching; following deduplication and screening, four studies were included in the final synthesis [6-9].

The database search identified 3,165 records across five databases (Scopus: n = 1,160; PubMed: n = 1,119; Web of Science: n = 430; Embase: n = 362; Cochrane CENTRAL: n = 94). Following automated deduplication via Covidence (n = 1,865 duplicates removed), 1,300 unique records were screened at the title and abstract level, of which 1,250 were excluded. Fifty full-text articles were sought for retrieval (all successfully obtained) and assessed for eligibility; 46 were excluded (wrong population: n = 17; wrong intervention: n = 24, including trials with non-injectable comparators, such as oral agents; wrong study design: n = 3; not retrievable: n = 2). Four studies met all inclusion criteria and were included in the final synthesis. The PRISMA 2020 flow diagram is presented below (Figure 1).

Study Characteristics

Four RCTs from the SUSTAIN clinical programme were included, with a combined total of 3,680 randomised participants across all treatment arms (trial-level N: SUSTAIN 3: n = 813; SUSTAIN 4: n = 1,089; SUSTAIN 7: n = 1,201; SUSTAIN 10: n = 577). For each pairwise analysis, only the semaglutide arm and the relevant active-comparator arm were extracted; where trials included multiple dose arms (SUSTAIN 4: three arms; SUSTAIN 7: four arms), the semaglutide 1.0 mg arm and the primary comparator arm were used for the 1.0 mg analyses, with the 0.5 mg arm reserved for sensitivity analyses. The total analysable population for the primary HbA1c analysis (semaglutide 1.0 mg vs. all comparators) was 2,705 participants (analysis-level N: SUSTAIN 3: n = 809; SUSTAIN 4: n = 720; SUSTAIN 7: n = 599; SUSTAIN 10: n = 577). This figure was derived as follows: SUSTAIN 3 (n = 809: semaglutide 1.0 mg n = 404 + exenatide ER n = 405); SUSTAIN 4 (n = 720: semaglutide 1.0 mg n = 360 + insulin glargine n = 360); SUSTAIN 7 (n = 599: semaglutide 1.0 mg n = 300 + dulaglutide 1.5 mg n = 299); SUSTAIN 10 (n = 577: semaglutide 1.0 mg n = 290 + liraglutide 1.2 mg n = 287). No arm was double-counted; the semaglutide 0.5 mg arms from SUSTAIN 4 (n = 362) and SUSTAIN 7 (n = 301), and the dulaglutide 0.75 mg arm from SUSTAIN 7 (n = 299), were excluded from the primary analysis and reserved for sensitivity analyses. All four trials enrolled insulin-naïve adults with T2DM inadequately controlled on OADs and compared once-weekly semaglutide with an active injectable comparator over 30-56 weeks. The key characteristics of the included studies are summarised in Table 1.

**Table 1:** Characteristics of the included studies. Semaglutide Unabated Sustainability in Treatment of Type 2 Diabetes (SUSTAIN) 3 [6], SUSTAIN 4 [7], SUSTAIN 7 [8], and SUSTAIN 10 [9] *QW = once weekly; QD = once daily; SU = sulfonylurea; TZD = thiazolidinedione; OAD = oral antidiabetic drug. Analysis-level N reflects only the semaglutide 1.0 mg arm and the primary comparator arm extracted for the pairwise analysis. SUSTAIN 4 (total randomised n = 1,089) and SUSTAIN 7 (total randomised n = 1,201) each included additional arms (semaglutide 0.5 mg and dulaglutide 0.75 mg, respectively) that were excluded from the primary analysis and reserved for the sensitivity analyses. The combined analysis-level population across all four trials was 2,705 participants.

Study (trial)	Comparator	Comparator dose	Sema 1.0 mg N	Comparator N	Analysis-level N	Duration (weeks)	Baseline HbA1c (%)	Background OAD
SUSTAIN 3 (Ahmann et al., 2017)	Exenatide ER	2.0 mg QW	404	405	809	56	8.3	Metformin ± SU ± TZD (1-2 OADs)
SUSTAIN 4 (Aroda et al., 2017)	Insulin glargine	Titrated (mean 29.2 IU/day)	360	360	720	30	8.2	Metformin ± SU
SUSTAIN 7 (Pratley et al., 2018)	Dulaglutide	1.5 mg QW	300	299	599	40	8.2	Metformin monotherapy (≥1500 mg/day)
SUSTAIN 10 (Capehorn et al., 2020)	Liraglutide	1.2 mg QD	290	287	577	30	8.2	Metformin ± SU ± SGLT-2i (1–3 OADs)

Risk of Bias

**Figure 2:**
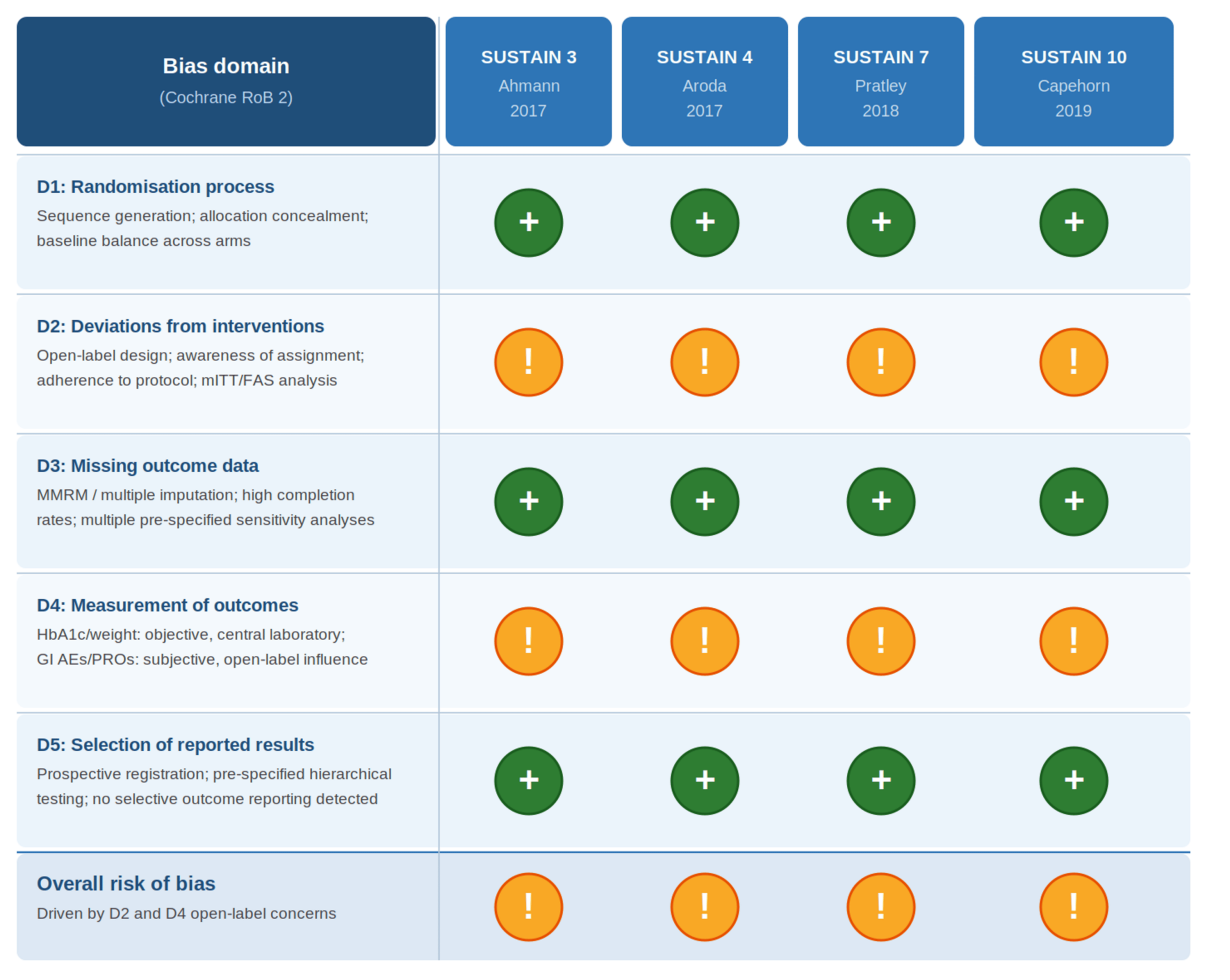
Cochrane Risk of Bias version 2 (RoB 2) traffic light plot for the included studies. D1–D5 represent the five RoB 2 domains. Green (+) = low risk; Orange (!) = some concerns. Overall risk of bias was judged as "some concerns" for all trials, driven by the open-label design (D2) and subjective outcome measurement concerns (D4) [6-9].

All four included trials were assessed using the Cochrane RoB 2 tool across five domains (Figure 2). Domain 1 (randomisation process) was rated as low risk in all trials, reflecting adequate sequence generation, allocation concealment, and baseline balance. Domain 2 (deviations from intended interventions) was rated as "some concerns" in all trials owing to their open-label designs, although mITT/FAS analyses were employed throughout. Domain 3 (missing outcome data) was rated as low risk in all trials, with high completion rates and pre-specified sensitivity analyses using MMRM and multiple imputation. SUSTAIN 3 had the highest treatment discontinuation rate across the four trials (approximately 20% in both arms), predominantly due to gastrointestinal (GI) adverse events with semaglutide and injection-site reactions with exenatide ER; all remaining trials achieved treatment completion rates of 82-91%, with trial completion rates of 94-99%. Domain 4 (measurement of the outcome) was rated as "some concerns" in all trials: HbA1c and body weight were measured objectively via a central laboratory, but GI adverse events and patient-reported outcomes are subject to open-label influence. Domain 5 (selection of the reported result) was rated as low risk in all trials, supported by prospective registration and pre-specified hierarchical testing with no selective outcome reporting detected. Overall risk of bias was judged as "some concerns" for all four trials, driven by the open-label design concerns in D2 and D4. This overall judgement is consistent with the phase 3 regulatory trial context. Importantly, the impact of open-label design is not uniform across outcomes: HbA1c and body weight were measured objectively via central laboratory and standardised assessments and are therefore minimally susceptible to performance or detection bias arising from unblinded treatment allocation. By contrast, subjective outcomes, including GI adverse event reporting, treatment discontinuation due to adverse events, and patient-reported tolerability, are more vulnerable to open-label influence, as the awareness of treatment assignment may affect both the threshold for reporting symptoms and the decision to discontinue. Readers should therefore interpret the safety and tolerability findings with greater caution than the primary glycaemic and weight outcomes.

Primary Outcome: HbA1c Reduction

**Figure 3:**
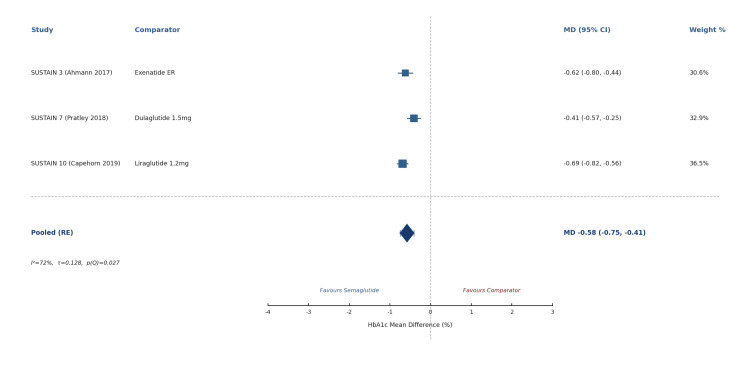
Forest plot of the HbA1c change (%). Semaglutide 1.0 mg versus other GLP-1 receptor agonists: SUSTAIN 3 [6], SUSTAIN 7 [8], SUSTAIN 10 [9]. RE = random-effects model. MD = mean difference

Semaglutide 1.0 mg versus other GLP-1 RAs: Pairwise meta-analysis of three trials comparing semaglutide 1.0 mg with other GLP-1 RAs (SUSTAIN 3: exenatide ER; SUSTAIN 7: dulaglutide 1.5 mg; SUSTAIN 10: liraglutide 1.2 mg) demonstrated that semaglutide produced a significantly greater reduction in HbA1c (MD -0.58%, 95% CI -0.75 to -0.41; I² = 72%; τ = 0.128; p(Q) = 0.027). All three constituent trials individually favoured semaglutide, with individual MDs ranging from -0.41% (versus dulaglutide 1.5 mg) to -0.69% (versus liraglutide 1.2 mg) (Figure 3). The 95% PI for this comparison was -2.549% to +1.396%. Because this interval crosses zero, it indicates that the true effect in a future head-to-head trial could favour either semaglutide or the comparator: the lower bound represents a clinically meaningful additional HbA1c reduction of 2.5 percentage points with semaglutide, while the upper bound represents a potential inferiority of 1.4 percentage points. This wide interval reflects substantial between-trial heterogeneity (I² = 72%) driven by differences in the comparator agent, background OAD therapy, and trial duration and means that claims of uniform HbA1c superiority across all GLP-1 RA comparators in future settings cannot be made with confidence on the basis of this evidence alone.

**Figure 4:**
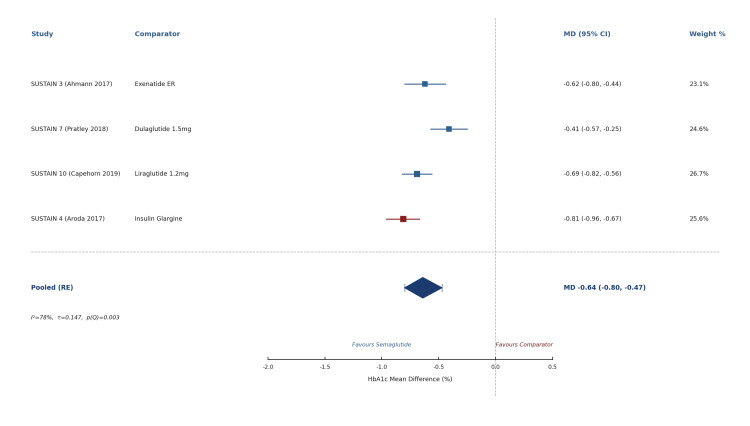
Forest plot of the HbA1c change (%). Semaglutide 1.0 mg versus all injectable comparators: Semaglutide Unabated Sustainability in Treatment of Type 2 Diabetes (SUSTAIN) 3 [6], SUSTAIN 4 [7], SUSTAIN 7 [8], SUSTAIN 10 [9]. Blue squares represent GLP-1 RA comparators; red square represents insulin glargine.

Semaglutide 1.0 mg versus all injectable comparators (combined analysis): When all four trials were pooled (including SUSTAIN 4 versus insulin glargine), the overall pooled effect further confirmed semaglutide superiority (MD -0.64%, 95% CI -0.80 to -0.47; I² = 78%; τ = 0.147; p(Q) = 0.003). The effect against insulin glargine (SUSTAIN 4) was the most pronounced (MD -0.81%, 95% CI -0.96 to -0.67), followed by liraglutide (MD -0.69%) and exenatide ER (MD -0.62%), with the most modest advantage versus dulaglutide 1.5 mg (MD -0.41%) (Figure 4). The 95% PI for the combined analysis was -1.363% to +0.091%, which crosses zero at the upper bound. This indicates that while the pooled estimate robustly favours semaglutide, a future trial drawn from this mixed-comparator evidence base could yield a result ranging from clinically meaningful superiority to a near-null or marginally unfavourable outcome; this reflects the substantial between-study variance introduced by pooling mechanistically disparate comparator classes (GLP-1 RAs and basal insulin). Stratified PIs by comparator class are therefore more informative for clinical interpretation than the overall pooled PI.

Secondary Outcomes

**Figure 5:**
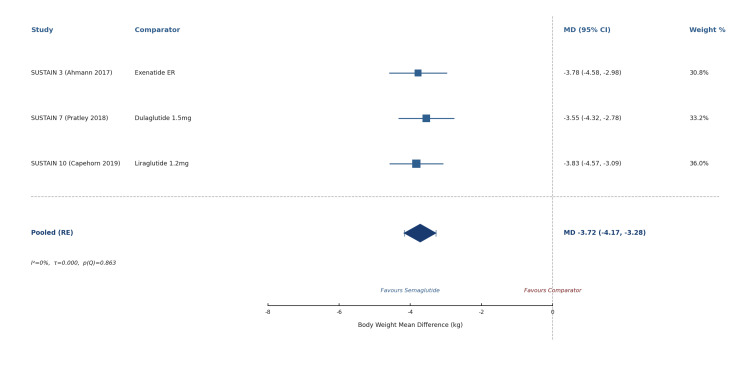
Forest plot of the body weight change (kg). Semaglutide 1.0 mg versus other GLP-1 receptor agonists: Semaglutide Unabated Sustainability in Treatment of Type 2 Diabetes (SUSTAIN) 3 [6], SUSTAIN 7 [8], SUSTAIN 10 [9].

**Figure 6:**
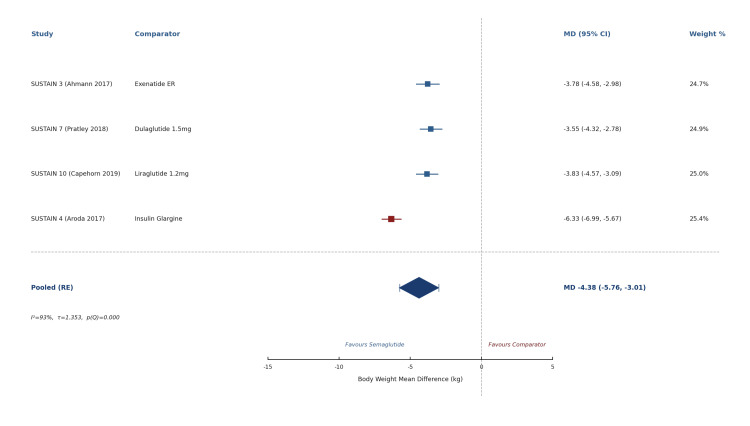
Forest plot of the body weight change (kg). Semaglutide 1.0 mg versus all injectable comparators: Semaglutide Unabated Sustainability in Treatment of Type 2 Diabetes (SUSTAIN) 3 [6], SUSTAIN 4 [7], SUSTAIN 7 [8], SUSTAIN 10 [9].

**Figure 7:**
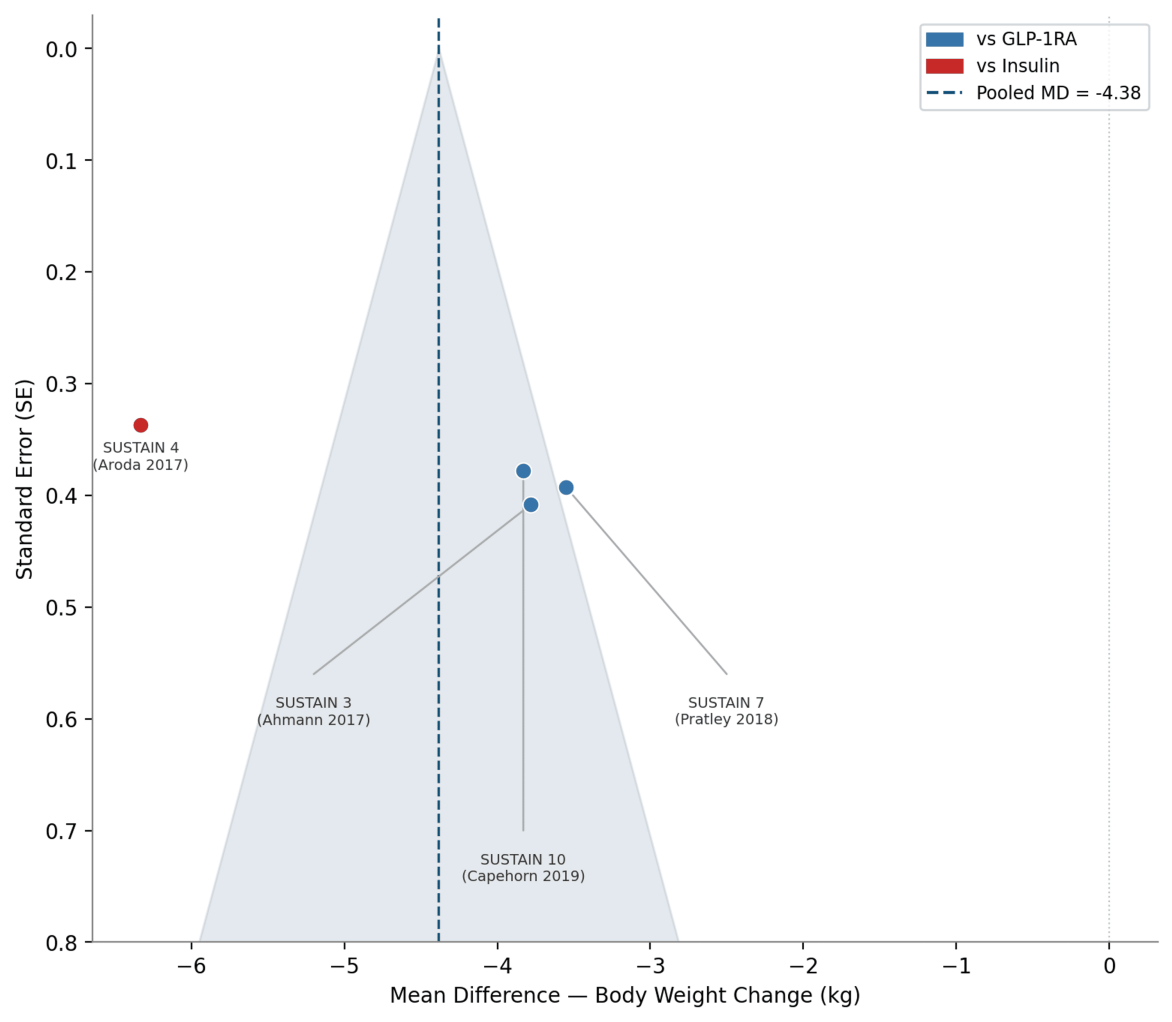
Funnel plot for the body weight change. All comparators (descriptive only; not interpreted as evidence of publication bias). Blue circles represent GLP-1 RA comparators; red circle represents insulin glargine. The pooled MD line is shown as a dashed vertical reference. Apparent asymmetry reflects comparator-class heterogeneity (insulin glargine versus GLP-1 RAs) rather than small-study effects.

Body weight: Semaglutide 1.0 mg produced significantly greater reductions in body weight compared with other GLP-1 RAs [MD -3.72 kg, 95% CI -4.17 to -3.28; I² = 0%; τ = 0.000; p(Q) = 0.863], with no significant heterogeneity across the three GLP-1 RA comparisons (Figure 5). The 95% PI was -6.60 kg to -0.84 kg, which remains entirely in the favourable direction, indicating that semaglutide is expected to produce superior weight reduction compared with other GLP-1 RAs across a range of future clinical settings. The weight advantage was more pronounced when insulin glargine was included in the pooled analysis: semaglutide reduced body weight by a mean of 4.38 kg more than all comparators combined (MD -4.38 kg, 95% CI -5.76 to -3.01; I² = 93%; τ = 1.353; p(Q) < 0.001], driven substantially by the SUSTAIN 4 result where insulin glargine was associated with weight gain (MD -6.33 kg, 95% CI -6.99 to -5.67) (Figure 6). The 95% PI for the combined analysis was -10.95 kg to +2.18 kg, which crosses zero at the upper bound. This wide interval, spanning from an approximately 11 kg advantage to a 2 kg disadvantage for semaglutide, appropriately tempers any claim of consistent weight superiority across all injectable comparators when these are pooled indiscriminately. The PI should be interpreted stratified by comparator class: when restricted to GLP-1 RA comparators, the PI is entirely favourable (-6.60 to -0.84 kg), supporting class-consistent weight superiority of semaglutide over other GLP-1 RAs specifically; however, when insulin glargine is included, the combined PI crossing zero indicates that the true weight differential in a future mixed-comparator setting cannot be reliably predicted from this evidence base. A funnel plot for the body weight change was generated for descriptive purposes (Figure 7). The apparent asymmetry, with SUSTAIN 4 (insulin glargine) occupying the left outlier position, should not be interpreted as evidence of publication bias; rather, it reflects the expected comparator-class heterogeneity between GLP-1 RA and basal insulin comparators. With only four included studies, funnel plot asymmetry cannot be reliably distinguished from between-study heterogeneity, and formal statistical testing for publication bias (e.g., Egger's test) is not appropriate with fewer than 10 studies per the Cochrane Handbook guidance.

**Figure 8:**
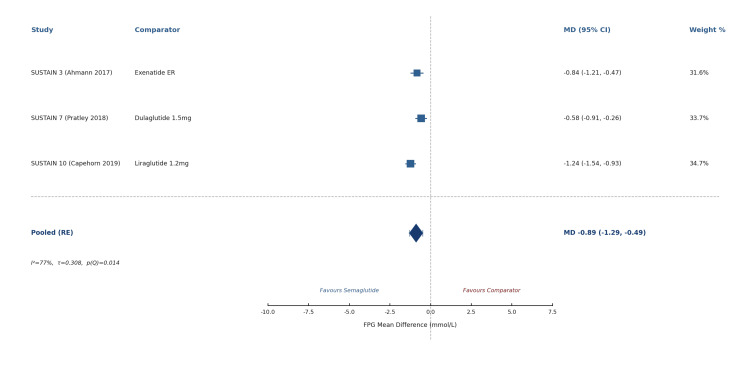
Forest plot of the fasting plasma glucose change (mmol/L). Semaglutide 1.0 mg versus other GLP-1 receptor agonists: Semaglutide Unabated Sustainability in Treatment of Type 2 Diabetes (SUSTAIN) 3 [6], SUSTAIN 7 [8], SUSTAIN 10 [9].

Fasting plasma glucose: Semaglutide significantly reduced fasting plasma glucose (FPG) compared with GLP-1 RA comparators (MD -0.89 mmol/L, 95% CI -1.29 to -0.49; I² = 77%; τ = 0.308; p(Q) = 0.014), with the greatest difference observed versus liraglutide 1.2 mg (MD -1.24 mmol/L, 95% CI -1.54 to -0.93) (Figure 8). The 95% PI for FPG versus GLP-1 RAs was -5.58 to +3.79 mmol/L, which crosses zero substantially in both directions. Because this interval includes values both below and above zero, it indicates that the true effect on FPG in a future trial could favour either semaglutide or the comparator, reflecting marked between-trial variability (I² = 77%) in fasting glucose effects driven by differences in comparator agent and background OAD therapy. The pooled estimate favours semaglutide, but the magnitude of FPG benefit cannot be reliably predicted for an individual future study context.

**Figure 9:**
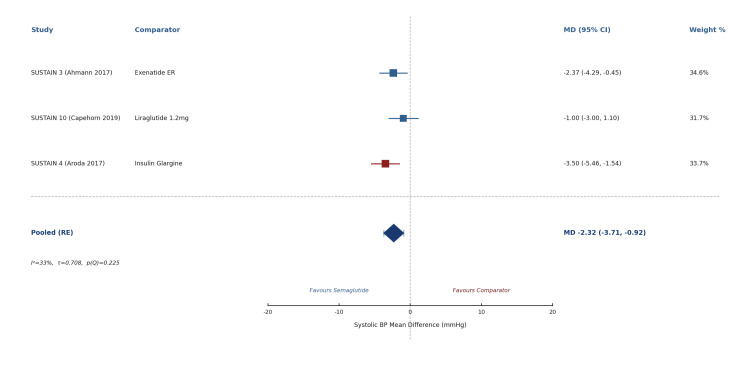
Forest plot of the systolic blood pressure change (mmHg). Semaglutide versus injectable comparators: Semaglutide Unabated Sustainability in Treatment of Type 2 Diabetes (SUSTAIN) 3 [6], SUSTAIN 4 [7], SUSTAIN 7 [8], SUSTAIN 10 [9].

Systolic blood pressure: Semaglutide produced a statistically significant reduction in systolic blood pressure (SBP) compared with all injectable comparators (MD -2.32 mmHg, 95% CI -3.71 to -0.92; I² = 33%; τ = 0.708; p(Q) = 0.225). The greatest SBP reduction was observed in SUSTAIN 4 versus insulin glargine (MD -3.50 mmHg, 95% CI -5.46 to -1.54) (Figure 9).

**Figure 10:**
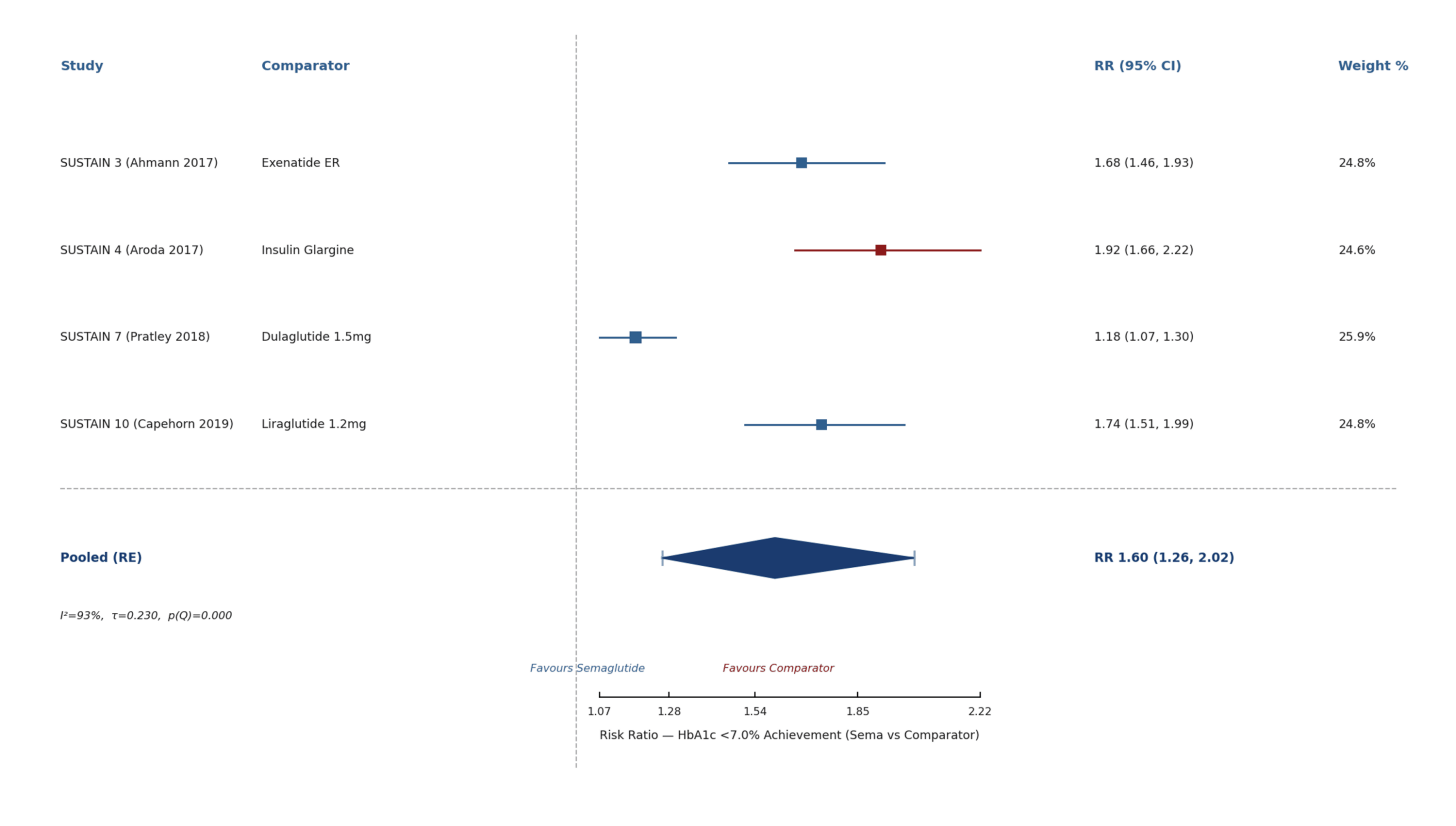
Forest plot of the HbA1c <7.0% achievement. Semaglutide versus all injectable comparators: Semaglutide Unabated Sustainability in Treatment of Type 2 Diabetes (SUSTAIN) 3 [6], SUSTAIN 4 [7], SUSTAIN 7 [8], SUSTAIN 10 [9]. Risk ratio.

HbA1c target achievement (<7.0%): Patients treated with semaglutide were significantly more likely to achieve the ADA HbA1c target of less than 7.0% compared with all injectable comparators (RR 1.60, 95% CI 1.26-2.02; I² = 93%; τ = 0.230; p(Q) < 0.001; 95% PI RR 0.52-4.87). Because the PI crosses 1.0 at the lower bound, it indicates that the true effect on the HbA1c target achievement in a future trial could favour either semaglutide or the comparator; the advantage observed in these four trials cannot be assumed to replicate in a different clinical context. This uncertainty reflects the very high between-study heterogeneity (I² = 93%) driven by differing baseline achievement rates and target achievement rates across comparator classes. Risk ratios were consistently greater than 1.0 across all individual trials, ranging from 1.18 (versus dulaglutide 1.5 mg) to 1.92 (versus insulin glargine) (Figure 10).

**Figure 11:**
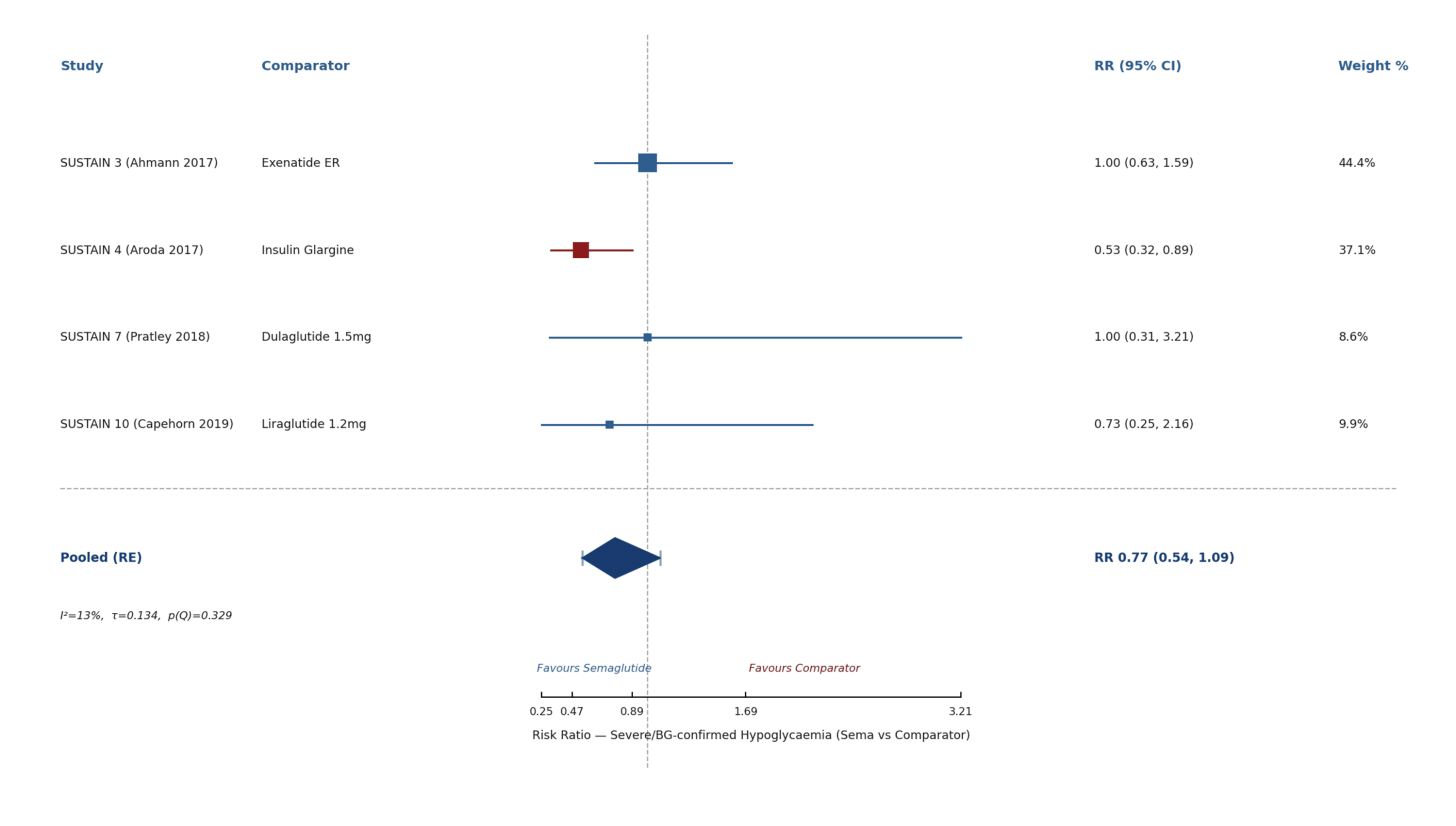
Forest plot of the hypoglycaemia risk (severe/BG-confirmed). Semaglutide versus all injectable comparators: Semaglutide Unabated Sustainability in Treatment of Type 2 Diabetes (SUSTAIN) 3 [6], SUSTAIN 4 [7], SUSTAIN 7 [8], SUSTAIN 10 [9]. Risk ratio.

Hypoglycaemia: Overall, the risk of severe or blood-glucose-confirmed hypoglycaemia was not significantly different between semaglutide and all injectable comparators in the pooled analysis (RR 0.77, 95% CI 0.54-1.09; I² = 13%; τ = 0.134; p(Q) = 0.329; 95% PI RR 0.53-1.12). The low heterogeneity and narrow PI for hypoglycaemia indicate that the finding of comparable hypoglycaemia risk between semaglutide and injectable comparators overall is relatively stable across study contexts, with the exception of the insulin glargine comparison (SUSTAIN 4) where semaglutide conferred a significant benefit. However, SUSTAIN 4 demonstrated a significantly lower hypoglycaemia risk with semaglutide compared with insulin glargine (RR 0.53, 95% CI 0.32-0.89), consistent with the known hypoglycaemia-sparing pharmacology of GLP-1 RAs. Hypoglycaemia rates were comparable between semaglutide and the GLP-1 RA comparators (Figure 11).

Gastrointestinal Adverse Events

**Figure 12:**
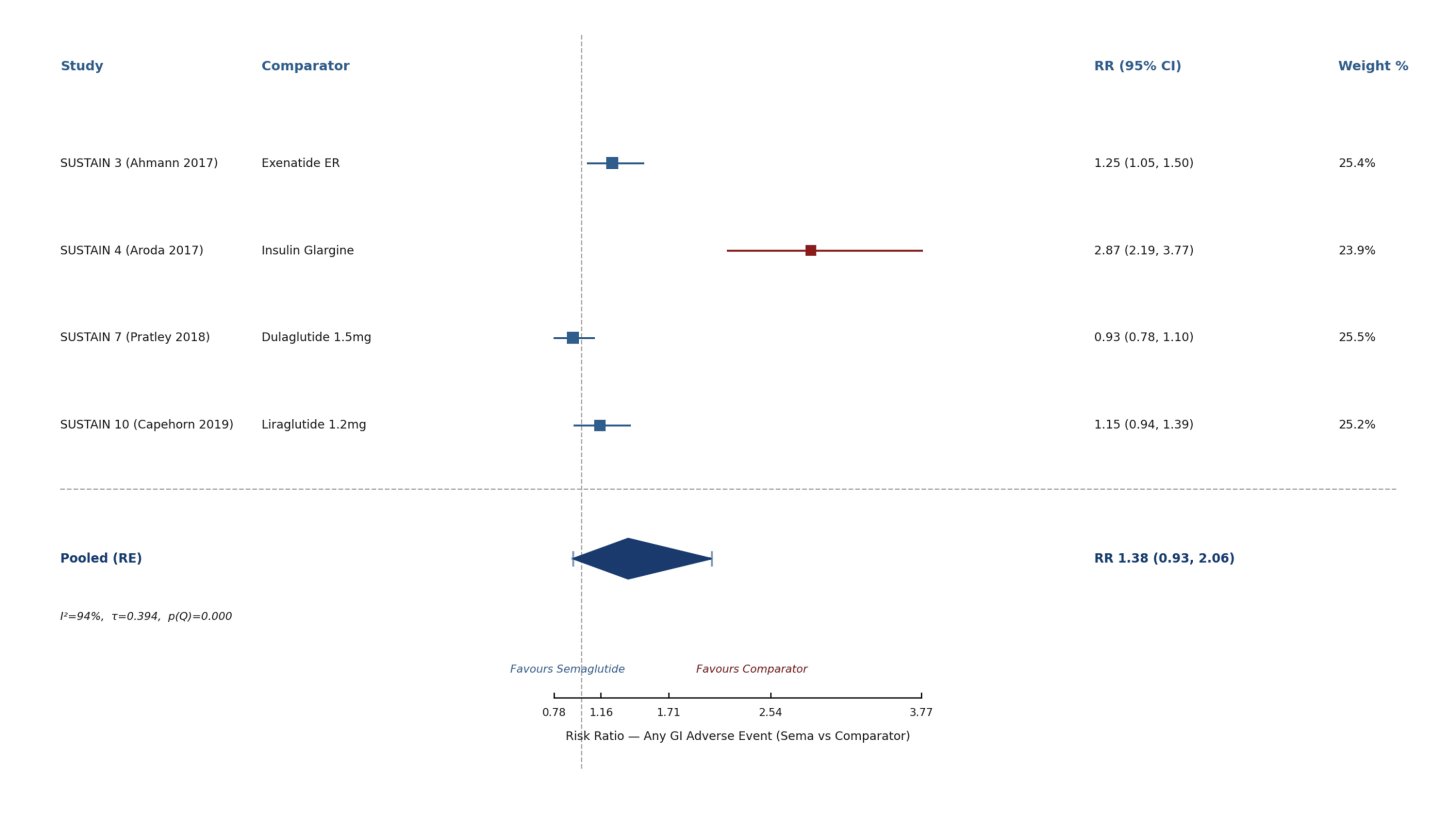
Forest plot of any gastrointestinal adverse event. Semaglutide versus all injectable comparators: Semaglutide Unabated Sustainability in Treatment of Type 2 Diabetes (SUSTAIN) 3 [6], SUSTAIN 4 [7], SUSTAIN 7 [8], SUSTAIN 10 [9]. Risk ratio.

Given the fundamentally different expected GI adverse-event profiles of basal insulin and GLP-1 receptor agonists, the pooled risk ratio across all comparators is of limited clinical interpretability and should not be emphasised. When stratified by comparator class, GI adverse event rates with semaglutide were substantially higher than with insulin glargine (RR 2.87, 95% CI 2.19-3.77; SUSTAIN 4), an expected pharmacological class effect reflecting the GI tolerability burden of GLP-1 RAs relative to basal insulin. By contrast, GI adverse event rates were not significantly different between semaglutide and other GLP-1 RA comparators (exenatide ER: RR 1.25, 95% CI 1.05-1.50; dulaglutide 1.5 mg: RR 0.93, 95% CI 0.78-1.10; liraglutide 1.2 mg: RR 1.15, 95% CI 0.94-1.39), indicating that semaglutide's GI profile is broadly comparable to other agents within its class. The overall pooled estimate (RR 1.38, 95% CI 0.93-2.06; I² = 94%) is driven entirely by the insulin glargine comparison and is not interpretable as a class-independent estimate of GI risk with semaglutide (Figure 12).

Treatment Discontinuation Due to Adverse Events

**Figure 13:**
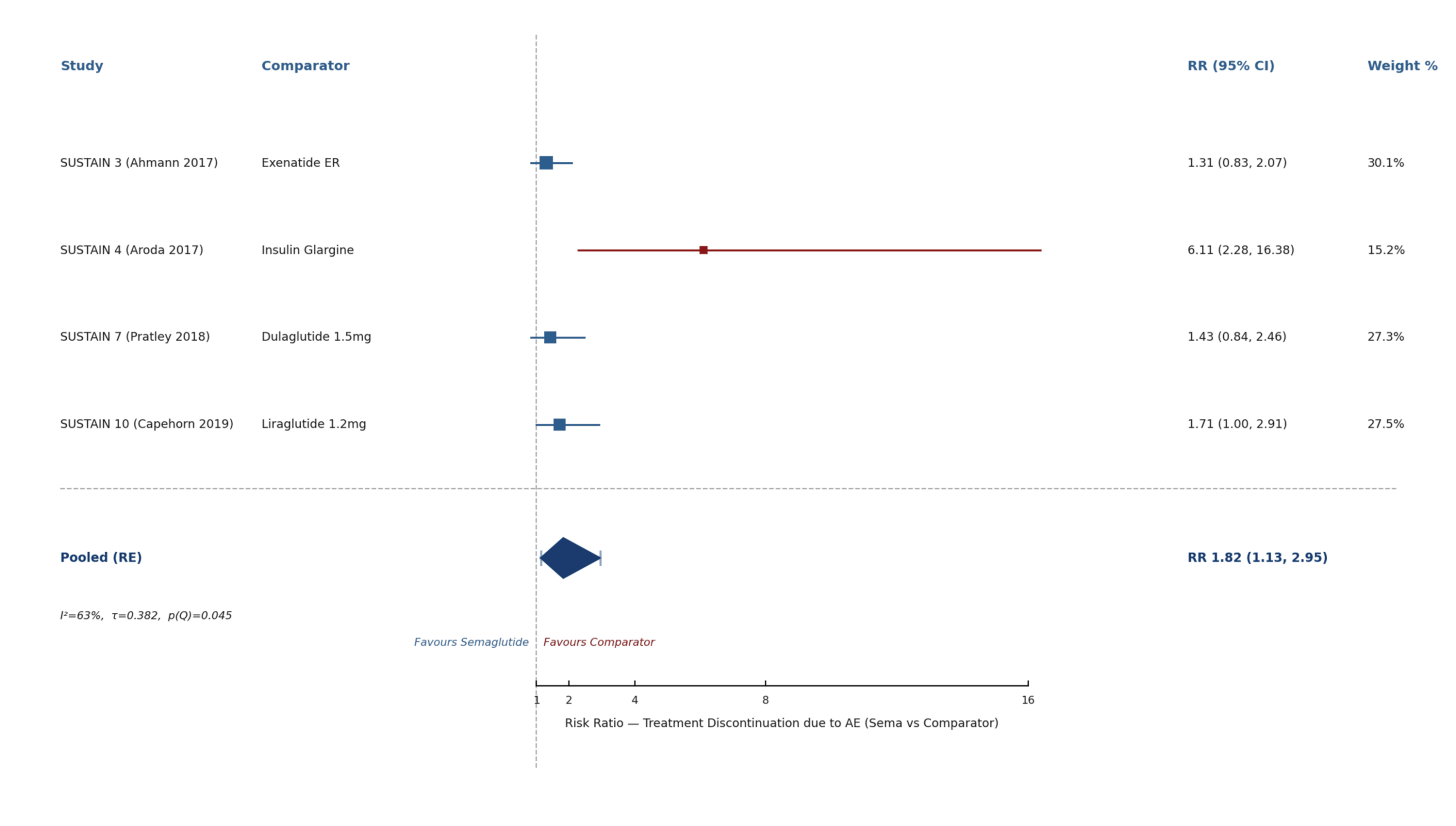
Forest plot of treatment discontinuation due to adverse events. Semaglutide versus all injectable comparators: Semaglutide Unabated Sustainability in Treatment of Type 2 Diabetes (SUSTAIN) 3 [6], SUSTAIN 4 [7], SUSTAIN 7 [8], SUSTAIN 10 [9]. Risk ratio.

Treatment discontinuation due to adverse events was significantly more frequent with semaglutide than comparators (RR 1.82, 95% CI 1.13-2.95; I² = 63%; τ = 0.382; p(Q) = 0.045; 95% PI RR 0.26-12.80). The wide PI, which includes values below 1.0 and above 10.0, reflects the extreme heterogeneity driven by SUSTAIN 4 (RR 6.11 vs. insulin glargine); among GLP-1 RA comparisons alone discontinuation rates were not significantly different, indicating that the pooled discontinuation signal is primarily a cross-class phenomenon, driven by SUSTAIN 4 (RR 6.11, 95% CI 2.28-16.38 vs. insulin glargine), reflecting the higher GI burden of GLP-1 RAs relative to insulin. Among GLP-1 RA comparisons, discontinuation rates showed a non-significant trend favouring comparators (Figure 13).

Outcomes Not Amenable to Meta-Analysis: Postprandial Glucose, Lipid Profile, and Heart Rate

Three pre-specified secondary outcomes - postprandial plasma glucose increment, lipid profile parameters, and heart rate - could not be pooled in formal meta-analysis owing to heterogeneous reporting formats, inconsistent use of standard errors versus standard deviations across trial publications, and insufficient studies per comparator class to generate stable pooled estimates. Individual trial data are summarised narratively below and presented in full in the supplementary data extraction file (see Appendix for the link; Sheets S6-S7).

Postprandial plasma glucose increment: Semaglutide 1.0 mg produced statistically significant reductions in the mean postprandial glucose increment compared with all four injectable comparators across all trials in which this outcome was assessed. In SUSTAIN 4, semaglutide 1.0 mg reduced the mean postprandial increment by an additional 0.65 mmol/L (95% CI 0.39-0.91; p < 0.0001) versus insulin glargine. In SUSTAIN 3, the additional reduction versus exenatide ER was 0.24 mmol/L (95% CI 0.04-0.44; p = 0.019). In SUSTAIN 7, the advantage over dulaglutide 1.5 mg was 0.30 mmol/L (95% CI 0.06-0.53; p = 0.013). In SUSTAIN 10, semaglutide 1.0 mg reduced the postprandial increment by a further 0.53 mmol/L (95% CI 0.28-0.77; p < 0.0001) versus liraglutide 1.2 mg. The directional consistency across all four trials indicates that semaglutide's postprandial glucose-lowering effect exceeds that of both basal insulin and other GLP-1 RAs; however, the magnitude of effect varied across comparator classes, and pooling was not considered appropriate given this clinical heterogeneity.

Lipid profile: Lipid data were reported at varying levels of granularity across the four trials, precluding formal meta-analysis. In SUSTAIN 4, semaglutide 1.0 mg produced statistically significant reductions in total cholesterol, LDL cholesterol, and triglycerides compared with insulin glargine, with free fatty acids as the only lipid fraction showing no significant between-group difference; C-reactive protein and plasminogen activator inhibitor-1 were also significantly reduced with both semaglutide doses versus insulin glargine. In SUSTAIN 3, semaglutide 1.0 mg significantly reduced VLDL cholesterol and triglycerides versus exenatide ER (both p<0.0001), with free fatty acids also significantly lower. In SUSTAIN 7, no clinically relevant differences in any lipid parameter were observed between semaglutide and dulaglutide at either dose level. In SUSTAIN 10, semaglutide 1.0 mg produced significantly greater improvements in total cholesterol and triglycerides versus liraglutide 1.2 mg. Taken together, the lipid data suggest a favourable profile for semaglutide compared with basal insulin and, to a lesser extent, versus other GLP-1 RAs, though the absence of standardised reporting and the lack of a common comparison node preclude quantitative synthesis.

Heart rate: A small but consistent increase in heart rate was observed with semaglutide across all trials, consistent with the known class effect of GLP-1 receptor agonists. In SUSTAIN 4, semaglutide 1.0 mg was associated with a mean increase of approximately 3.1 bpm versus baseline, representing a statistically significant between-group difference versus insulin glargine (ETD +3.19 bpm; p < 0.0001). In SUSTAIN 3, the heart rate increase with semaglutide 1.0 mg was 2.1 bpm, with a non-significant between-group difference versus exenatide ER (ETD +1.03 bpm; p = 0.097). In SUSTAIN 7, semaglutide 1.0 mg was associated with a statistically significant increase versus dulaglutide 1.5 mg (ETD +1.55 bpm, 95% CI 0.15-2.95; p = 0.030). In SUSTAIN 10, the between-group difference versus liraglutide 1.2 mg was not statistically significant (ETD -1.36 bpm; p = 0.056). The clinical relevance of these small heart rate increases is uncertain and was not associated with a higher rate of cardiac adverse events in any trial.

Cardiovascular Outcomes

None of the four included trials - SUSTAIN 3, 4, 7, and 10 - were designed or powered to assess cardiovascular outcomes. All four trials enrolled insulin-naïve adults with T2DM who were inadequately controlled on OADs, a population with relatively low baseline cardiovascular risk; adjudicated major adverse cardiovascular events (MACE: cardiovascular death, non-fatal myocardial infarction, non-fatal stroke) were not pre-specified primary or secondary endpoints in any of the four trials, and event rates in these short-duration phase 3 studies (30-56 weeks) were too low and inconsistently reported to support meta-analysis. While cardiac adverse events were included in overall safety reporting, these were not adjudicated or systematically characterised across all four trials in a standardised manner. Notably, semaglutide's cardiovascular benefit was established through the dedicated SUSTAIN 6 cardiovascular outcomes trial, which enrolled a high-cardiovascular-risk population already receiving insulin or GLP-1 RAs in many cases - a population fundamentally different from those in the four trials included in this review, and which therefore did not meet our eligibility criteria (see the *Limitations* section). The absence of cardiovascular outcome data from this meta-analysis represents a substantive limitation for extrapolating findings to patients with established cardiovascular disease, in whom the cardiovascular risk-benefit profile of injectable therapy selection is a primary clinical consideration. Clinicians treating patients with T2DM and established atherosclerotic cardiovascular disease should refer to the dedicated cardiovascular outcomes trial literature for this question, as the present review cannot address it.

Sensitivity Analyses

**Figure 14:**
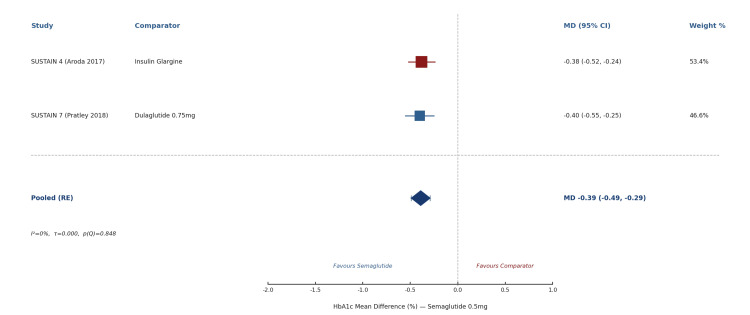
Sensitivity analysis. HbA1c reduction with semaglutide 0.5 mg versus comparators: Semaglutide Unabated Sustainability in Treatment of Type 2 Diabetes (SUSTAIN) 4 [7], SUSTAIN 7 [8].

**Figure 15:**
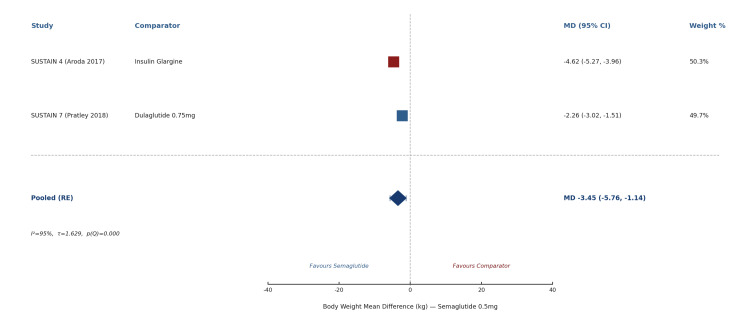
Sensitivity analysis. Body weight change with semaglutide 0.5 mg versus comparators: Semaglutide Unabated Sustainability in Treatment of Type 2 Diabetes (SUSTAIN) 4 [7], SUSTAIN 7 [8].

**Figure 16:**
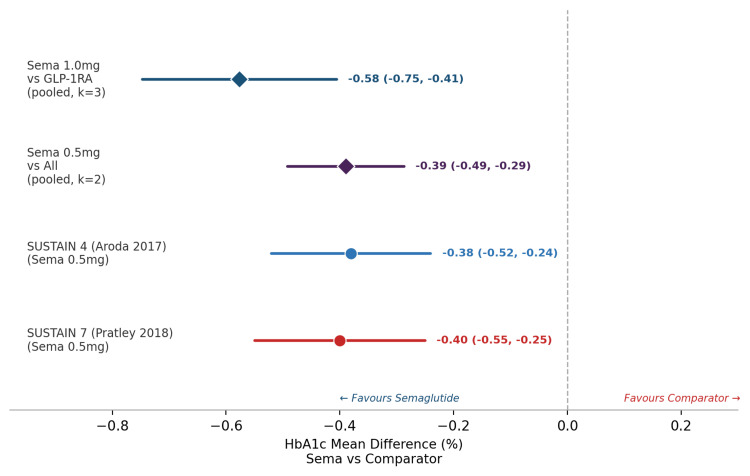
Dose-sensitivity overview. HbA1c reduction with semaglutide 1.0 mg (pooled vs. GLP-1 RAs: Semaglutide Unabated Sustainability in Treatment of Type 2 Diabetes (SUSTAIN) 3 [6], SUSTAIN 7 [8], SUSTAIN 10 [9]) versus semaglutide 0.5 mg (pooled vs. all comparators: SUSTAIN 4 [7], SUSTAIN 7 [8]) and individual SUSTAIN arms (0.5 mg).

Semaglutide 0.5 mg: Sensitivity analysis restricted to the semaglutide 0.5 mg dose arms (SUSTAIN 4: versus insulin glargine; SUSTAIN 7: versus dulaglutide 0.75 mg) confirmed consistent superiority in HbA1c reduction, albeit with a smaller magnitude of effect than the 1.0 mg dose (pooled MD -0.39%, 95% CI -0.49 to -0.29; I² = 0%; p(Q) = 0.848) (Figure 14). For body weight, results also favoured semaglutide 0.5 mg (MD -3.45 kg, 95% CI -5.76 to -1.14), although heterogeneity was high (I² = 95%), reflecting the disparate weight effects versus insulin glargine versus dulaglutide (Figure 15). Figure 16 presents a comparative dose-sensitivity overview across analyses.

Heterogeneity Assessment

**Figure 17:**
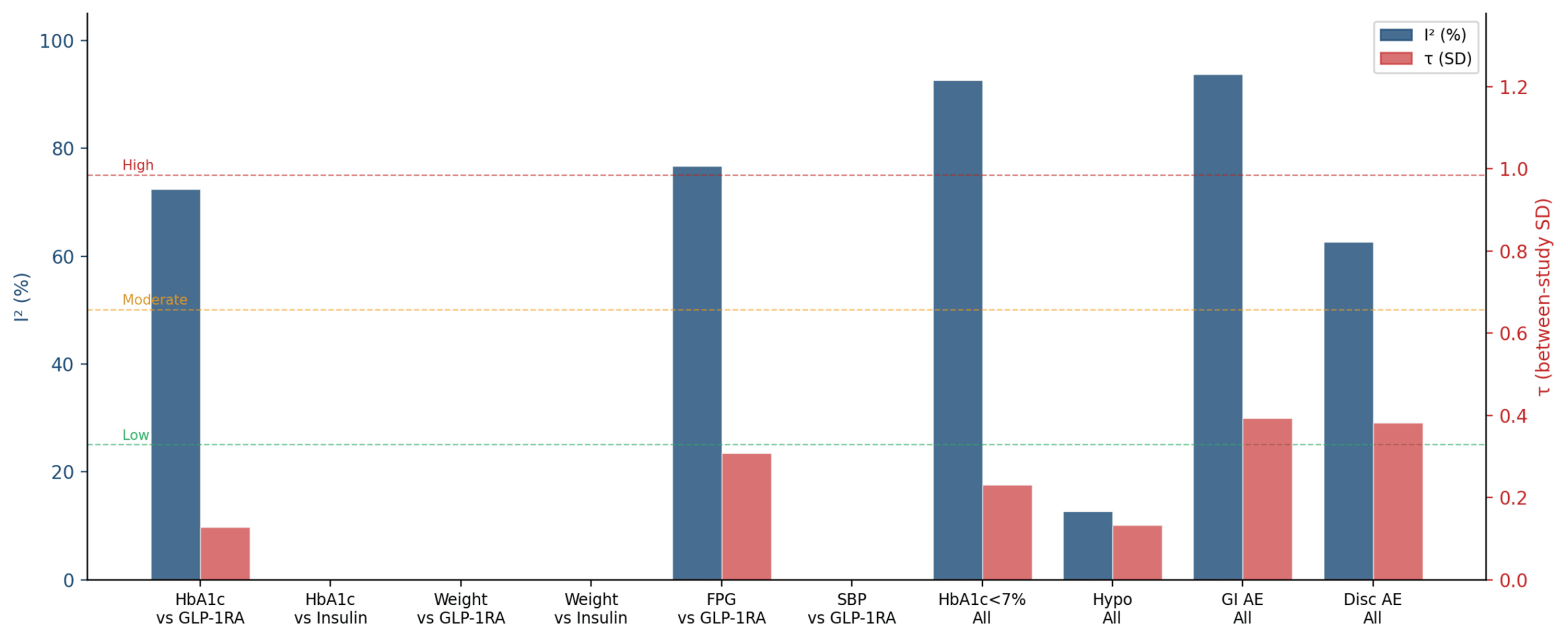
Summary of heterogeneity statistics (I² and τ) across all meta-analyses. Semaglutide Unabated Sustainability in Treatment of Type 2 Diabetes (SUSTAIN) 3 [6], SUSTAIN 4 [7], SUSTAIN 7 [8], SUSTAIN 10 [9]. Dashed horizontal lines indicate approximate I² reference values of 40% (not important), 60% (moderate), and 90% (considerable), interpreted contextually per Cochrane Handbook guidance [16].

The heterogeneity profile across all meta-analyses is summarised in Figure 17. Heterogeneity was absent or low for body weight change versus GLP-1 RAs (I² = 0%), HbA1c versus insulin glargine (I² = 0%), and systolic blood pressure (I² = 33%). Moderate-to-high heterogeneity was observed for HbA1c versus GLP-1 RAs (I² = 72%), FPG versus GLP-1 RAs (I² = 77%), and HbA1c target achievement across all comparators (I² = 93%). Extreme heterogeneity was present for GI adverse events (I² = 94%) and body weight across all comparators (I² = 93%), both attributable to the inclusion of the insulin glargine comparison arm, which represents a mechanistically distinct comparator. This comparator-driven heterogeneity was anticipated and underscores the clinical rationale for performing class-stratified and overall analyses separately.

Sensitivity Analysis: Exclusion of SUSTAIN 4 (Insulin Glargine Comparator)

Given the concern that insulin glargine in SUSTAIN 4 may have been under-titrated relative to contemporary standards (mean final dose 29.2 IU/day; mean fasting SMPG 7.1 mmol/L at week 30, compared with the guideline target of ≤5.6 mmol/L), a pre-specified sensitivity analysis was conducted excluding SUSTAIN 4 entirely and restricting all pooled estimates to the three GLP-1 RA comparator trials (SUSTAIN 3, 7, and 10). This analysis isolates the semaglutide-versus-GLP-1 RA comparison from any potential inflation attributable to suboptimal basal insulin management. The results are presented below and should be considered alongside the primary analysis when interpreting the insulin glargine findings.

When SUSTAIN 4 was excluded, semaglutide 1.0 mg continued to demonstrate significant superiority over GLP-1 RA comparators for all continuous primary and secondary outcomes. For HbA1c reduction, the pooled estimate across the three GLP-1 RA trials was MD -0.58% (95% CI -0.75 to -0.41; I² = 72%), identical to the stratified GLP-1 RA result reported in the *Semaglutide 1.0 mg Versus Other GLP-1 RAs* section, confirming that the primary HbA1c superiority is not dependent on the insulin glargine comparison. For body weight, the exclusion of SUSTAIN 4 yielded a pooled MD of -3.72 kg (95% CI -4.17 to -3.28; I² = 0%; PI -6.60 to -0.84 kg), with no heterogeneity and a PI entirely in the favourable direction - in marked contrast to the combined analysis including insulin glargine (MD -4.38 kg; PI -10.95 to +2.18 kg). For HbA1c target achievement (<7.0%), the GLP-1 RA-only pooled RR was 1.51 (95% CI 1.16-1.96; I² = 93%), remaining statistically significant but attenuated compared with the all-comparator estimate (RR 1.60), with the high residual heterogeneity reflecting genuine between-comparator variation within the GLP-1 RA class. Hypoglycaemia risk was comparable between semaglutide and GLP-1 RA comparators in the absence of the insulin glargine trial (RR 0.96, 95% CI 0.64-1.44; I² = 0%), consistent with the class-equivalent mechanism of action. A formal best/worst-case sensitivity analysis estimating what HbA1c outcomes would have been with optimally titrated insulin glargine (fasting glucose ≤6.1 mmol/L) was not feasible from published aggregate data, as individual patient-level titration data and the dose-response relationship for this specific cohort were not available in the public domain. The GLP-1 RA-only analysis presented here represents the available empirical proxy for quantifying the influence of the insulin glargine comparison on the pooled estimates.

Publication Bias

**Figure 18:**
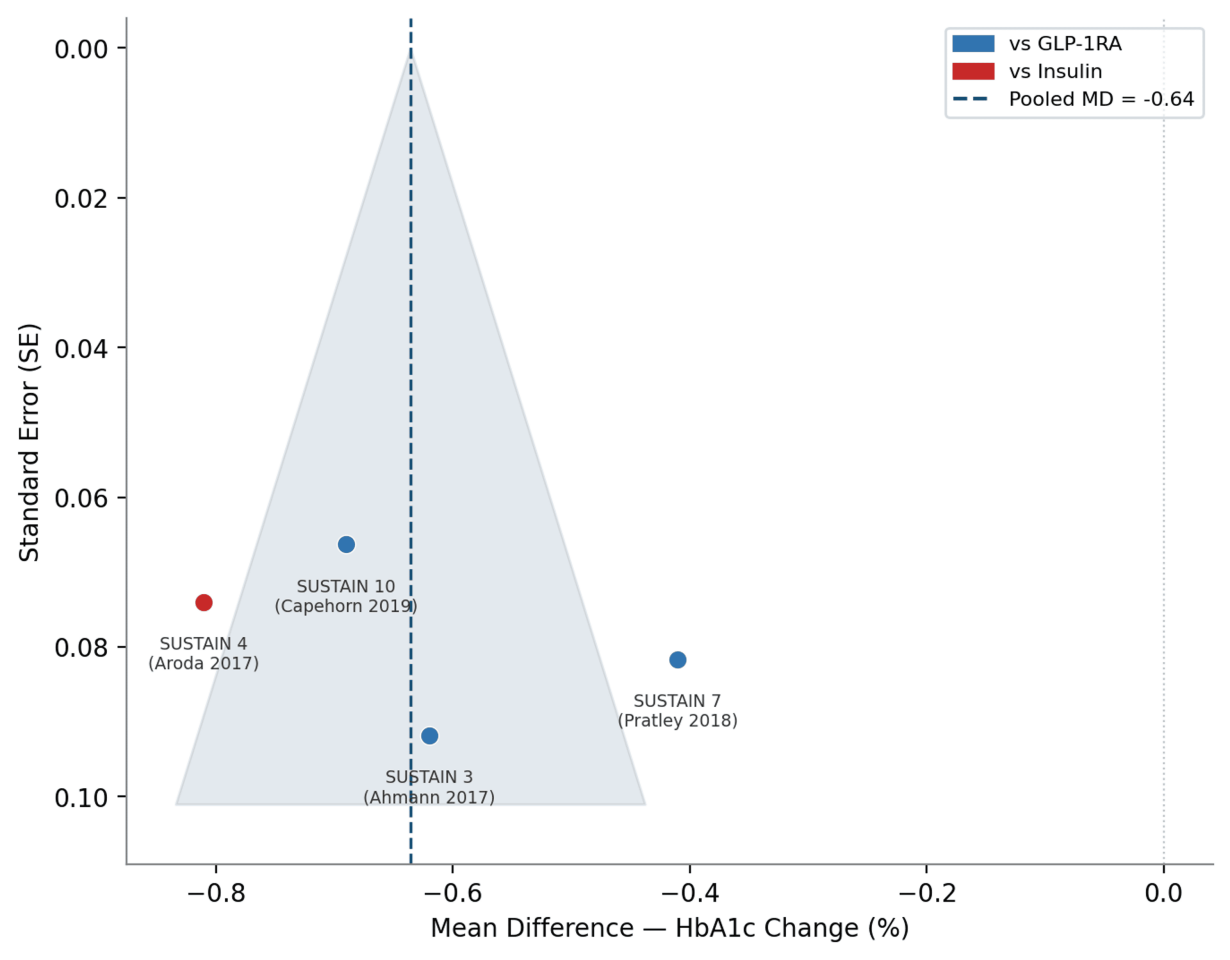
Funnel plot for the HbA1c change All comparators: Semaglutide Unabated Sustainability in Treatment of Type 2 Diabetes (SUSTAIN) 3 [6], SUSTAIN 4 [7], SUSTAIN 7 [8], SUSTAIN 10 [9]. Blue circles represent GLP-1 RA comparators; red circle represents insulin glargine.

Given the small number of included studies (n = 4), formal statistical testing for publication bias was not performed. Visual inspection of the funnel plot for HbA1c change (Figure 18) demonstrated broad symmetry, with studies distributed around the pooled effect estimate. The outlying position of SUSTAIN 4 in the body weight funnel plot (Figure 5) reflects genuine clinical heterogeneity due to the mechanistically different comparator (insulin glargine) rather than publication bias. This asymmetry should not be interpreted as indicative of selective outcome reporting or small-study effects, as discussed in the *Body Weight *section. The comprehensiveness of the search strategy, including trial registry searches and absence of language restrictions, mitigates concerns about publication bias in this focused evidence base.

Certainty of Evidence (GRADE)

The GRADE summary of findings is presented in Table 2. A uniform risk of bias downgrade (-1) was applied to all outcomes, reflecting the open-label design of all four contributing trials ("some concerns" on RoB 2 domains D2 and D4) and the 100% industry-sponsored evidence base, both of which represent meaningful risk of bias concerns at the body-of-evidence level that apply equally across all outcomes. This consistent application corrects the selective downgrading present in earlier iterations of this table. Certainty was therefore rated as low for HbA1c reduction versus GLP-1 RAs (-1 risk of bias; -1 serious heterogeneity, I² = 72%) and low for HbA1c reduction versus all comparators (-1 risk of bias; -1 serious heterogeneity, I² = 78%). Body weight reduction versus GLP-1 RAs was rated as moderate (-1 risk of bias only; I² = 0%, no heterogeneity downgrade), representing the highest certainty rating in this review. Hypoglycaemia risk and systolic blood pressure were each rated as moderate (-1 risk of bias; low or absent heterogeneity). Outcomes with very serious heterogeneity received two downgrades - risk of bias plus very serious inconsistency - yielding low certainty for HbA1c target achievement (I² = 93%), GI adverse events (I² = 94%), body weight versus all comparators (I² = 93%), and treatment discontinuation (I² = 63%).

**Table 2:** GRADE Summary of Findings Studies contributing to each analysis: SUSTAIN 3 [6], SUSTAIN 4 [7], SUSTAIN 7 [8], SUSTAIN 10 [9]. CI = confidence interval; MD = mean difference; RR = risk ratio; GLP-1 RA = glucagon-like peptide-1 receptor agonist; GI = gastrointestinal; BP = blood pressure; AE = adverse event. GRADE certainty: ⊕⊕⊕⊕ High; ⊕⊕⊕○ Moderate; ⊕⊕○○ Low; ⊕○○○ Very low.

Outcome	Studies (N)	Effect estimate (95% CI)	I² (%)	Certainty	Key downgrade reasons
HbA1c reduction vs. all comparators	4 (2,705)	MD −0.64% (−0.80, −0.47)	78%	⊕⊕○○ Low	Risk of bias [open-label design; single-sponsor evidence base]; serious heterogeneity [I²=78%]
HbA1c reduction vs. GLP-1 RAs	3 (1,985)	MD −0.58% (−0.75, −0.41)	72%	⊕⊕○○ Low	Risk of bias [open-label design; single-sponsor evidence base]; serious heterogeneity [I²=72%]
Body weight vs. GLP-1 RAs	3 (1,985)	MD −3.72 kg (−4.17, −3.28)	0%	⊕⊕⊕○ Moderate	Single-sponsor evidence base; open-label design
Body weight vs. all comparators	4 (2,705)	MD −4.38 kg (−5.76, −3.01)	93%	⊕⊕○○ Low	Risk of bias (open-label design; single-sponsor evidence base); very serious heterogeneity (I²=93%)
HbA1c <7.0% achievement	4 (2,705)	RR 1.60 (1.26, 2.02)	93%	⊕⊕○○ Low	Risk of bias (open-label design; single-sponsor evidence base); very serious heterogeneity (I²=93%)
Hypoglycaemia (all)	4 (2,705)	RR 0.77 (0.54, 1.09)	13%	⊕⊕⊕○ Moderate	Risk of bias (open-label design; single-sponsor evidence base)
GI adverse events	4 (2,705)	RR 1.38 (0.93, 2.06)	94%	⊕⊕○○ Low	Risk of bias (open-label design; single-sponsor evidence base); very serious heterogeneity (I²=94%)
Treatment discontinuation (AE)	4 (2,705)	RR 1.82 (1.13, 2.95)	63%	⊕⊕○○ Low	Risk of bias (open-label design; single-sponsor evidence base); serious heterogeneity (I²=63%)
Systolic BP	3 (1,985)	MD −2.32 mmHg (−3.71, −0.92)	33%	⊕⊕⊕○ Moderate	Risk of bias (open-label design; single-sponsor evidence base)

Discussion

Principal Findings

This systematic review and meta-analysis of four head-to-head RCTs from the SUSTAIN programme demonstrates that once-weekly semaglutide 1.0 mg is superior to all evaluated injectable antidiabetic therapies for glycaemic control and body weight reduction in insulin-naïve adults with T2DM inadequately controlled on OADs. Semaglutide produced a clinically and statistically significant additional HbA1c reduction of 0.64 percentage points (95% CI 0.47-0.80) compared with all comparators, with the magnitude of superiority ranging from 0.41 percentage points versus dulaglutide 1.5 mg to 0.81 percentage points versus insulin glargine. Hypoglycaemia risk was significantly lower with semaglutide compared with insulin glargine, while GI adverse events were more frequent. The sensitivity analysis with semaglutide 0.5 mg confirmed a dose-dependent but consistent pattern of glycaemic superiority.

An important caveat to the consistently favourable pooled estimates is provided by the 95% PIs, which quantify the expected range of true effects in a future study drawn from the same evidence base. For HbA1c reduction versus GLP-1 RAs, the PI was −2.549% to +1.396%, indicating that while the pooled estimate robustly favours semaglutide, a future head-to-head trial could plausibly yield a result ranging from clinically meaningful superiority to marginal inferiority. Similarly, the PI for FPG versus GLP-1 RAs crossed zero in both directions (-5.58 to +3.79 mmol/L), and the combined body weight PI also crossed zero (-10.95 to +2.18 kg), the latter driven entirely by the mechanistically heterogeneous inclusion of insulin glargine as a comparator. For HbA1c target achievement (RR PI 0.52-4.87) and GI adverse events (RR PI 0.21-9.32), PIs were extremely wide, reflecting very high between-study heterogeneity. By contrast, body weight reduction versus GLP-1 RAs specifically had a PI entirely in the favourable range (-6.60 to -0.84 kg), and hypoglycaemia risk PI was narrow and stable (RR 0.53-1.12). These PIs must be interpreted honestly rather than dismissed. For outcomes where the PI crosses zero (HbA1c versus GLP-1 RAs: -2.549% to +1.396%; FPG vs. GLP-1 RAs: -5.58 to +3.79 mmol/L; body weight combined: -10.95 to +2.18 kg; HbA1c target achievement: RR 0.52-4.87), the true effect in a future trial or specific clinical setting could favour either semaglutide or the comparator - a finding that appropriately limits the strength of inferential conclusions. The pooled estimates represent the best available central tendency across these four trials, and the directional consistency of individual trial results provides some reassurance, but the between-study variance is sufficiently large that the magnitude of benefit in any future study cannot be reliably predicted. The most clinically robust PI findings are the body weight advantage versus GLP-1 RAs (PI entirely favourable) and the stable hypoglycaemia risk (PI narrow, excluding meaningful excess risk). For all other outcomes with PI crossing zero, clinicians should interpret the pooled estimates as directional signals - consistent in sign across trials, but uncertain in magnitude - rather than as precise quantitative predictions applicable to individual clinical contexts.

Glycaemic Efficacy in Context

The magnitude of additional HbA1c reduction achieved with semaglutide 1.0 mg is clinically relevant. An HbA1c reduction of 0.5 percentage points over background therapy has been widely adopted as a threshold for clinically meaningful benefit [4]. The pooled effect of -0.58% versus other GLP-1 RAs and -0.64% versus all injectable comparators substantially exceeds this threshold, supporting the clinical relevance of semaglutide 1.0 mg as a highly efficacious HbA1c-lowering GLP-1 RA, with moderate certainty evidence given the single-sponsor evidence base. This superiority is particularly notable in the direct comparison with liraglutide 1.2 mg (MD -0.69%), as both are GLP-1 RAs with similar mechanisms of action, with the additional efficacy likely attributable to semaglutide's longer half-life (approximately 165 hours vs. 13 hours for liraglutide), higher albumin binding, and greater receptor occupancy at the weekly dose [18]. The greater HbA1c reduction versus insulin glargine - a comparator that is titrated to fasting glucose targets - reflects semaglutide's ability to address both fasting and postprandial hyperglycaemia simultaneously, a pharmacodynamic advantage over basal insulin monotherapy.

The finding that 60% more patients achieved the HbA1c target of less than 7.0% with semaglutide compared with other injectable therapies (RR 1.60, 95% CI 1.26-2.02) is of particular clinical importance. Achievement of individualised HbA1c targets is the primary goal of pharmacological escalation in T2DM, and the substantially higher proportion of patients reaching this target with semaglutide has direct implications for treatment sequencing and intensification decisions in clinical practice.

Body Weight and Cardiometabolic Benefits

The weight reduction achieved with semaglutide warrants particular emphasis. Against other GLP-1 RAs - all of which are weight-neutral or weight-reducing - semaglutide produced an additional 3.72 kg reduction (95% CI 3.28-4.17; I² = 0%), with no heterogeneity, indicating a highly consistent and robust class-within-class effect. Against insulin glargine, the differential was even more striking (−6.33 kg vs. insulin glargine alone), as basal insulin is typically associated with weight gain of 1-3 kg. In the context of a condition where obesity drives both pathophysiology and cardiovascular risk, this dual metabolic benefit - simultaneous glycaemic control and weight reduction - represents a fundamental clinical advantage over insulin-based approaches [4].

The additional reduction in systolic blood pressure of 2.32 mmHg (95% CI 0.92-3.71) observed with semaglutide compared with all injectable therapies further augments its cardiometabolic profile. Although modest in absolute terms, reductions of this magnitude are associated with reduced cardiovascular event rates at a population level, and are consistent with the pleiotropic vasculoprotective effects of GLP-1 RAs observed in cardiovascular outcomes trials [10,19].

Safety Profile: Hypoglycaemia and Gastrointestinal Events

The significantly lower hypoglycaemia risk with semaglutide versus insulin glargine (RR 0.53, 95% CI 0.32-0.89) reflects the glucose-dependent mechanism of GLP-1 RA action, which does not stimulate insulin secretion under euglycaemic conditions. This safety advantage is clinically relevant, particularly in primary care settings, where fear of hypoglycaemia represents a recognised barrier to insulin initiation and intensification [5]. The comparable hypoglycaemia rates among GLP-1 RA comparisons (RR 0.77, overall; 95% CI 0.54-1.09) indicate that semaglutide does not confer an additional hypoglycaemia risk within the class.

The higher rate of GI adverse events with semaglutide compared with insulin glargine (RR 2.87, 95% CI 2.19-3.77) is expected, given the mechanistic class difference and is consistent with the GI adverse event profile of all GLP-1 RAs. Nausea, vomiting, and diarrhoea are typically transient, peaking in the dose-escalation phase, and are manageable with gradual up-titration. The corresponding higher treatment discontinuation rate versus insulin glargine (RR 6.11) in SUSTAIN 4 reflects this GI burden, although absolute discontinuation rates in semaglutide arms across the SUSTAIN trials ranged from 4% to 10%. Importantly, when compared with other GLP-1 RAs, GI event rates and discontinuation rates were not significantly different, indicating that the overall safety signal is attributable to the insulin comparator driving heterogeneity, not an intrinsically worse tolerability of semaglutide within class.

Comparison With Existing Systematic Reviews

This review extends previous evidence syntheses in several important respects. Zaccardi et al. (2016) conducted a network meta-analysis (NMA) comparing once-weekly GLP-1 RAs but predated SUSTAIN 7 and SUSTAIN 10, limiting their analysis to exenatide ER and dulaglutide as comparators [11]. Witkowski et al. (2018) performed a systematic review, and the NMA was restricted to patients on one to two OADs and did not include direct comparisons with basal insulin [12]. Andreadis et al. (2018) assessed the efficacy and safety of semaglutide across both placebo-controlled and active-controlled trials but included mixed background therapy populations without restricting to insulin-naïve OAD-failure patients, and predated the publication of SUSTAIN 10 [13]. The present review is, to our knowledge, the first comprehensive pairwise meta-analysis to synthesise RCT evidence from all four major head-to-head SUSTAIN trials simultaneously - incorporating exenatide ER, dulaglutide, liraglutide, and insulin glargine as comparators - in a single, prospectively registered analysis restricted to insulin-naïve patients inadequately controlled on OADs. The inclusion of insulin glargine comparison data is particularly novel, providing cross-class evidence that directly informs the GLP-1 RA-versus-insulin decision at the point of injectable escalation.

Limitations

Several limitations of this review must be acknowledged.

First, the small number of eligible studies (n = 4) limits statistical power for several secondary outcomes and prevents formal publication bias testing.

Second, all included trials were sponsored by Novo Nordisk, the manufacturer of semaglutide, which may introduce industry sponsorship bias; however, as no independent head-to-head trials were identified, these represent the complete available evidence base.

Third, the open-label designs of all SUSTAIN trials introduce a potential for performance bias, although objective biochemical outcomes reduce this concern for the primary endpoint. Furthermore, the influence of open-label design is outcome-dependent: while objective laboratory outcomes (HbA1c, body weight, and FPG) are minimally affected by knowledge of treatment assignment, subjective outcomes, including GI adverse event reporting and treatment discontinuation due to adverse events, are more susceptible to performance and detection bias in unblinded trials. The higher rates of GI adverse events and discontinuation observed with semaglutide versus insulin glargine (SUSTAIN 4) may therefore partly reflect ascertainment bias rather than a true pharmacological difference.

Fourth, and critically, the SUSTAIN 4 insulin glargine comparison is subject to a potential systematic bias from comparator under-titration. Insulin glargine titration in SUSTAIN 4 was conducted at investigator's discretion without a dedicated titration committee; the mean final insulin dose was 29.2 IU/day, and the mean fasting self-monitored plasma glucose (SMPG) at week 30 was 7.1 mmol/L. Contemporary guidelines and dedicated titration trials (e.g., TITRATE, BEGIN trials) target fasting glucose ≤5.6 mmol/L with optimally titrated basal insulin, suggesting that the insulin glargine arm in SUSTAIN 4 was meaningfully under-titrated by modern standards. This matters because SUSTAIN 4 contributes the largest individual effect sizes in the entire meta-analysis: HbA1c MD −0.81% and body weight MD −6.33 kg. If insulin glargine had been titrated to a fasting glucose target of ≤5.6 mmol/L, a meaningful additional HbA1c reduction in the comparator arm would be expected, narrowing the observed difference. While the exact magnitude of this attenuation cannot be quantified without individual patient-level titration data - precluding a formal best/worst-case sensitivity analysis - published data from dedicated insulin titration trials suggest that optimally titrated glargine achieves HbA1c reductions approximately 0.2-0.4 percentage points greater than sub-optimally titrated regimens. Applying this range to the SUSTAIN 4 comparator arm would reduce the observed HbA1c advantage of semaglutide to approximately 0.41-0.61 percentage points, which, while still clinically meaningful (≥0.5% threshold), would substantially attenuate the pooled estimate when SUSTAIN 4 is included. The weight differential versus insulin glargine (MD −6.33 kg) is less affected by titration adequacy, as weight gain with insulin is largely a class effect independent of achieved glucose target. Readers are directed to Section 3.6.3, which presents a pre-specified sensitivity analysis excluding SUSTAIN 4 entirely and restricting pooled estimates to the GLP-1 RA comparator trials (SUSTAIN 3, 7, 10). The findings from that analysis - HbA1c MD -0.58% (95% CI -0.75 to -0.41), body weight MD -3.72 kg (95% CI -4.17 to -3.28) - represent the most conservative available estimate of semaglutide's advantage, free from potential insulin titration confounding, and are considered the most appropriate basis for comparing semaglutide against other GLP-1 RAs. The HbA1c advantage versus insulin glargine specifically should be interpreted with explicit acknowledgement that it may be partially inflated by sub-optimal comparator titration.

Fifth, no head-to-head trials exist connecting the individual comparator agents to each other; a connected evidence network with a common comparator node would allow a proper NMA in future updates.

Sixth, the absence of head-to-head trials comparing semaglutide against fixed-ratio combination therapies (IDegLira and iGlarLixi) is a genuine limitation of this review's scope. Although this absence reflects the current state of the published evidence base rather than a search or methodological failure, it means this review cannot answer how semaglutide compares against fixed-ratio combinations - an injectable escalation option that is clinically relevant and guideline-endorsed for patients in whom simultaneous GLP-1 RA and basal insulin effects are desired. The findings of this review do not apply to patients for whom fixed-ratio combinations are the primary treatment consideration.

Seventh, cardiovascular outcome data are not available from this meta-analysis. The four included trials were short-duration phase 3 glycaemic efficacy studies enrolling low-cardiovascular-risk OAD-failure patients; adjudicated MACE was not a pre-specified endpoint in any trial, and event rates were too low and inconsistently reported for meta-analysis. This represents a substantive limitation for patients with established atherosclerotic cardiovascular disease or high cardiovascular risk, in whom injectable therapy selection is informed by cardiovascular outcomes trial data rather than glycaemic efficacy alone (see Section 3.5.9 for further detail).

Eighth, outcome data on health-related quality of life and patient-reported outcomes were not consistently reported across trials and could not be meta-analysed.

Finally, all SUSTAIN trials utilised OAD background therapies predominantly including metformin with or without sulfonylureas; caution is warranted in extrapolating findings to patients on SGLT2 inhibitor or DPP-4 inhibitor backgrounds, which are increasingly prevalent in contemporary practice. In addition, a complete list of excluded full-text articles with individual reasons for exclusion was not retained in retrievable form following the screening process and could not be provided as a supplementary table; this represents a reporting limitation acknowledged in response to peer review, and complete screening records will be maintained in accordance with PRISMA 2020 requirements in future reviews.

A further and important methodological limitation is the absence of fully independent dual-reviewer screening and risk of bias assessment. The registered PROSPERO protocol specified two independent reviewers; however, both reviewers (F.A.A. and U.R.) are co-authors with prior familiarity with all four included trials, constituting a formal deviation from the protocol. The extent to which this deviation may have introduced bias cannot be fully excluded. Several contextual factors are noted without prejudice to this uncertainty: (i) RoB 2 domain judgements across all four trials were reached by consensus between the two co-authors, rather than by independent parallel assessment; (ii) the primary outcome (HbA1c change) is an objective biochemical measure assessed by central laboratory, which reduces - but does not eliminate - the potential for subjective reviewer influence on domain-level judgements; (iii) the evidence base comprises four well-known, publicly registered phase 3 trials with transparently reported outcomes; and (iv) the absence of recorded disagreements across all 20 domain-study combinations may reflect shared prior familiarity with the trials rather than genuine independent convergence. Readers should interpret all RoB 2 ratings with awareness of this limitation, and should not assume that the domain-level judgements are free from the influence of reviewer familiarity with the included trials. Future updates of this review should involve at least one reviewer with no prior authorship involvement in the included trials to fully satisfy the dual-reviewer independence standard.

Several notable trials from the SUSTAIN programme were identified during the search and considered for inclusion but excluded on pre-specified eligibility grounds; these are reported here transparently to assist interpretation of the evidence base. SUSTAIN 1 (semaglutide versus placebo, treatment-naïve patients) was excluded because the comparator was not an active injectable therapy. SUSTAIN 2 (semaglutide vs. sitagliptin) was excluded because sitagliptin is an oral agent, not an injectable therapy. SUSTAIN 5 (semaglutide as an add-on to basal insulin) was excluded because the study population was already on insulin at baseline, violating the insulin-naïve criterion. SUSTAIN 6 (cardiovascular outcomes trial) was excluded at full-text review under the wrong population: the trial enrolled patients with high cardiovascular risk, many of whom were already on insulin or had advanced disease beyond the OAD-failure population specified in our PICO. SUSTAIN 8 (semaglutide versus canagliflozin) was identified in the database search and excluded at full-text review under the wrong intervention, as canagliflozin is an oral SGLT-2 inhibitor and not an injectable antidiabetic therapy; this exclusion is subsumed within the wrong intervention category (n = 24) in the PRISMA flow diagram. PIONEER 4 (oral semaglutide versus injectable dulaglutide) was identified in the database search and excluded at full-text review because the intervention was oral semaglutide, not once-weekly subcutaneous semaglutide as required by our PICO; although the comparator (dulaglutide 0.75 mg) was injectable, the formulation difference precludes direct inclusion in a review restricted to subcutaneous semaglutide. Finally, of the 24 studies excluded for wrong intervention, none were trials of subcutaneous once-weekly semaglutide: these exclusions comprised trials of other GLP-1 receptor agonists (dulaglutide, liraglutide, exenatide, lixisenatide, albiglutide) evaluated without a semaglutide arm, trials of oral semaglutide formulations, and trials of subcutaneous semaglutide in populations already receiving insulin therapy.

Clinical Implications

The findings from this systematic review and pairwise meta-analysis have several implications for clinical practice, interpreted in the context of the evidence certainty ratings and methodological limitations described above. Among the injectable options directly compared in head-to-head RCTs to date, semaglutide 1.0 mg consistently produced greater HbA1c and body weight reductions than exenatide ER, insulin glargine, dulaglutide, and liraglutide, with a significantly lower hypoglycaemia risk than basal insulin. These findings are directionally consistent with the 2022 ADA/EASD consensus framework, which recommends GLP-1 RAs as the first injectable option in the absence of insulin-requiring indications, with preference for agents with proven cardiovascular benefit [4]. Clinicians considering injectable intensification in insulin-naïve patients may find the comparative efficacy and weight data from these trials clinically informative when individualising treatment decisions, particularly for patients where weight reduction and hypoglycaemia avoidance are priorities. However, given that all evidence derives from single-sponsor open-label trials and that GRADE certainty is moderate-to-low for several outcomes, these findings should inform rather than dictate clinical decision-making, and should be considered alongside cost-effectiveness, patient preference, local formulary availability, and individual comorbidity profile. The higher rate of GI adverse events with semaglutide compared with insulin is an expected class effect; standardised dose escalation schedules can help minimise early discontinuation.

## Conclusions

This systematic review and pairwise meta-analysis of four direct head-to-head RCTs suggests that once-weekly subcutaneous semaglutide 1.0 mg is associated with greater reductions in HbA1c and body weight compared with the active injectable comparators evaluated - exenatide extended-release, insulin glargine, dulaglutide, and liraglutide - in insulin-naïve adults with T2DM inadequately controlled on OADs. These findings should be interpreted with caution, given low-to-moderate certainty of evidence, substantial heterogeneity for several outcomes, a small number of contributing trials, and prediction intervals that cross the null for HbA1c reduction versus GLP-1 RAs, body weight versus all comparators, FPG, and HbA1c target achievement. Across the four trials, semaglutide was associated with additional HbA1c reductions of 0.41-0.81 percentage points beyond comparators, a higher proportion of patients achieving the HbA1c less than 7.0% target, body weight reductions of 2.3-6.3 kg greater than comparators, and lower systolic blood pressure, with a hypoglycaemia risk significantly lower than basal insulin. The consistency of directional findings across all four trials provides supportive evidence, but the magnitude of benefit should not be assumed to generalise beyond the populations and comparators studied. The principal safety trade-off is a higher rate of transient gastrointestinal adverse events compared with insulin, an expected pharmacological class effect.

These findings should be interpreted within their methodological context: all four trials were sponsored by Novo Nordisk, none were blinded, the evidence base comprises only four studies, precluding formal network comparisons, and GRADE certainty ratings range from moderate to low for several key outcomes. Prediction intervals for several outcomes cross zero - including HbA1c versus GLP-1 RAs, FPG, body weight combined, and HbA1c target achievement, indicating that the true effect in a future trial or specific clinical context could favour either semaglutide or the comparator and that the pooled estimates should be interpreted as directional signals rather than precise quantitative predictions. Within these constraints, the available direct comparative evidence consistently favours semaglutide 1.0 mg across glycaemic, weight, and hypoglycaemia outcomes. Importantly, this review does not provide evidence comparing semaglutide to fixed-ratio combination therapies (IDegLira, iGlarLixi), which represent a clinically relevant alternative injectable escalation strategy; its findings do not apply to patients for whom these combinations are the primary therapeutic consideration. Equally, this review does not address the cardiovascular outcome profile of semaglutide relative to active injectable comparators, as the included trials were not designed to detect CV events; clinicians managing patients with established cardiovascular disease should consult the dedicated cardiovascular outcomes trial literature for this question. Future evidence should incorporate independent trials, fixed-ratio combination comparators, cardiovascular outcome data, patient-reported outcomes, and a properly connected network meta-analysis as the evidence base matures.

## Appendices

Data extraction form

Link: https://docs.google.com/spreadsheets/d/1xmas5IzvGG6DAez_AKYb7HfClD2ySqpi/edit?usp=share_link&ouid=108014130722505860695&rtpof=true&sd=true
